# How the Acute Psychosocial Stress Response Ends: A Post-Stressor Transition Model of Glucocorticoid Feedback, Vagal Re-Engagement, and Frontolimbic Context Updating

**DOI:** 10.3390/ijms27209047

**Published:** 2026-10-12

**Authors:** Congcong Liu, Ronglian Zheng, Yihan Wu, Meng Zhang

**Affiliations:** 1 Department of Psychology, Henan Medical University, Xinxiang 453003, China; cc_uestc@outlook.com (C.L.);; 2 Mental Illness and Cognitive Neuroscience Key Laboratory of Xinxiang, Henan Medical University, Xinxiang 453003, China

**Keywords:** acute psychosocial stress, stress recovery, glucocorticoid receptor, FKBP5, vagally mediated heart rate variability, frontolimbic circuitry, perseverative cognition, temporal coordination, resilience

## Abstract

Objective stressor offset does not mark a common onset of recovery: cardiovascular activation may be waning while cortisol is still rising and threat-related cognition persists. This structured narrative review integrates molecular, autonomic, neural, cognitive, and immune evidence on the post-stressor interval, primarily in adult psychosocial-stress paradigms. We propose the Post-Stressor Transition Model (PSTM) as an operational extension of existing recovery and allostatic accounts, not as a newly discovered biological system. Three regulatory functions organize the synthesis: updating whether threat remains current, changing cardiac autonomic influence, and regulating continuing hypothalamic–pituitary–adrenocortical output. Glucocorticoid receptor signaling, catecholamines, endocannabinoids, oxytocin, and vasopressin contribute across these functions. The framework distinguishes objective offset from system-specific extrema, physiological resolution from demonstrated adaptation, and shared timing from directional coupling. Eight propositions specify outcomes, temporal windows, competing explanations, and prespecified effect bounds. Measurement recommendations address cortisol nonresponse, respiratory influences on heart rate variability, assessment reactivity, and multirate sampling. Hormonal state and development modify candidate parameters rather than define fixed response types. No universal recovery threshold or clinically validated transition profile is claimed. The model is useful only if its additional parameters are identifiable, improve independent prediction, and withstand phase-specific experimental tests.

## 1. Introduction: The Missing Biology After Stressor Offset

Acute stress mobilizes neural, endocrine, autonomic, metabolic, and immune resources to meet immediate demands. Its adaptive value depends on two linked capacities: generating a response of appropriate magnitude and curtailing or reorganizing that response as conditions change. Weak mobilization can compromise performance and safety; persistence after the challenge has passed can disrupt sleep, cognition, social engagement, tissue maintenance, and later adaptation. This dual requirement lies at the core of allostasis and of models that place the brain both upstream and downstream of stress physiology [1,2,3,4].

The Trier Social Stress Test and related social-evaluative paradigms have generated a substantial literature on cortisol, cardiovascular activity, affect, cognition, and individual differences [5,6,7,8,9]. Response magnitude and recovery have long been recognized as distinct targets; their conceptual separation is not new [10]. Baseline-to-peak change, maximum concentration, and area under the curve remain useful, but combine secretion, transport, peak timing, decline, and observation-window length in different ways [11,12]. The unresolved problem is how to connect these trajectories to specific regulatory processes after the challenge ends.

The difficulty is most apparent at objective stressor offset. Cardiac activity may be declining while salivary cortisol has not yet peaked and inflammatory changes remain undetectable. An apparent cross-system delay can therefore arise from ordinary kinetics rather than a regulatory failure. Conversely, persistent threat appraisal can sustain activity after external demands have ceased. A useful account must distinguish these alternatives rather than infer a common recovery state from elapsed task time.

Recent studies justify treating response generation and post-response regulation as related but separable. In peak-adjusted models, cortisol reactivity and recovery carried non-redundant information, although recovery did not consistently outperform reactivity as an index of long-term stress or health [13]. Multitrajectory analyses likewise reveal partially dissociable endocrine, autonomic, and affective patterns rather than a single continuum from low to high response [14,15,16]. In a 2026 trajectory study, limbic and prefrontal features contributed differently to cortisol and affective patterns, further indicating that acute stress comprises coupled processes with distinct dynamics [17].

This review examines acute psychological and psychosocial stress, with adult laboratory paradigms as its principal application. It does not attempt an exhaustive life-span review or a synthesis of cellular, exercise, thermal, or metabolic stress. Developmental studies and animal mechanisms are included where they test the limits of adult inference or explain processes inaccessible to direct human manipulation. The Post-Stressor Transition Model (PSTM) is proposed within these boundaries.

The framework has three commitments. It retains both the objective end of the challenge and each system’s own peak or nadir as temporal anchors. It distinguishes a physiological return toward a reference state from adaptation demonstrated by an independent behavioral or prospective outcome. It then compares independent recovery, shared-input accounts, and directional coupling using post-offset perturbations and out-of-sample prediction. These commitments extend, rather than replace, established recovery research; Section 7 states the specific differences from peak-adjusted, multi-trajectory, allostatic, and neurovisceral accounts.

## 2. Scope, Definitions, and Evidence Identification

Adult social-evaluative and uncontrollable-stress paradigms provide the main evidence base. The interval of interest extends from objective stressor offset through minutes to several hours, with repeated-stress studies informing habituation and sensitization. Animal and cellular studies are used to evaluate mechanisms, not to establish human recovery thresholds. Childhood, adolescence, and older age are considered explicitly as limits on transfer in Section 8.1.

Fear extinction and safety learning provide mechanistic comparisons but are not interchangeable with recovery from social-evaluative stress. Chronic stress, psychiatric symptoms, adversity, sleep, and reproductive hormones are considered chiefly as modifiers of baseline state, response gain, timing, or coupling. The review does not claim exhaustive coverage of every mediator or a validated taxonomy of stress-related disorders.

PubMed/MEDLINE was the principal and only bibliographic database directly consulted. Backward and forward citation tracking supplemented database identification; linked full texts were used to examine methods and interpretation. The initial evidence search covered the literature available through 10 August 2026, with targeted revision-stage searches in September 2026 on additional mediators, repeated inflammatory sampling, development, hormonal state, respiratory measurement, cortisol nonresponse, and statistical identification. This is a structured narrative review for mechanistic integration, not a systematic review or meta-analysis. No pooled effects or exhaustive search yield are claimed.

Evidence labels in Table 1 are qualitative judgments based on five considerations: study design and control of confounding, independent replication, temporal resolution, specificity of the measure to the proposed mechanism, and direct relevance to post-stressor change. “Strong mechanistic” denotes convergent experimental support for a component mechanism, not proof of the complete human model. “Moderate” denotes convergent but heterogeneous or incompletely specific human evidence; “emerging” denotes limited or mainly indirect evidence; and “insufficient” denotes absence of adequate validation for the stated use. Labels are not GRADE ratings, numerical scores, or independently adjudicated certainty estimates.

### 2.1. Stressor Offset, System-Specific Peaks, and Re-Equilibration

Objective stressor offset, t_0_, is the timed end of the external challenge; continuing observation, evaluation, or uncertainty must be recorded separately. A system-specific extremum is its peak or nadir within a prespecified episode window. Reactivity describes the deviation associated with mobilization, whereas recovery describes post-extremum movement toward a relevant reference state. An extremum can precede t_0_; its signed offset-relative timing must not be forced to zero. A peak at the final sample leaves recovery unobserved, and an absent measurable response leaves amplitude-normalized recovery undefined. Re-equilibration includes stabilization at a contextually appropriate level rather than necessarily at the initial laboratory mean.

A baseline is not a fixed physiological zero. Circadian phase, anticipatory stress, posture, respiration, recent activity, food intake, sleep, medication, and the laboratory context can shift the reference state. Nor is complete return to a pre-task mean always the most adaptive endpoint. A transient overshoot may reflect active regulation, whereas an apparently rapid return may be achieved through a qualitatively different state. The strongest designs therefore estimate trajectories relative to repeated pre-stressor observations and report both return-to-reference and stability after return.

Concurrent concordance concerns association at the same time, after accounting for the direction and scale of each measure; opposite-signed cortisol and vmHRV changes can still be coordinated. Temporal coordination concerns their relation across time. Neither a zero-lag correlation nor a delay alone identifies causal influence. Adaptation requires a separate outcome criterion, specified in Section 7.3.

**Figure 1:**
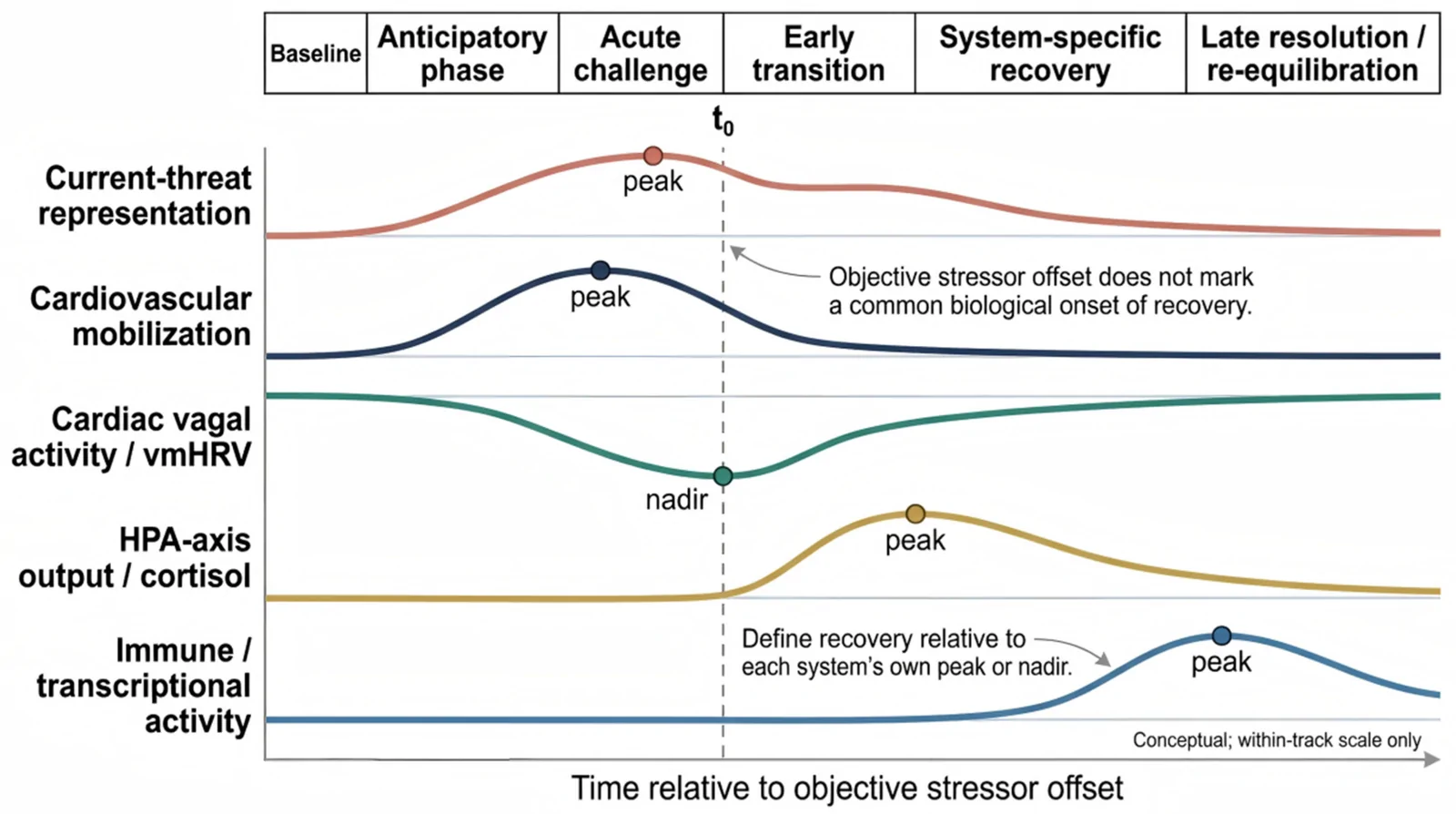
Conceptual temporal architecture of acute psychosocial stress and the post-stressor transition. The external challenge ends at t_0_, but current-threat representation, cardiovascular mobilization, cardiac vagal activity, HPA-axis output, and delayed immune or transcriptional activity follow different delays and time constants. System-specific recovery is defined relative to each system’s own peak or nadir rather than assumed to begin at task offset. Curves are conceptual and do not represent empirical means, normative values, or a fixed causal chain. Abbreviations: HPA, hypothalamic–pituitary–adrenocortical; vmHRV, vagally mediated heart rate variability.

Figure 1 illustrates why cardiac decline, the cortisol peak, and delayed immune activity must not be assigned one common recovery onset. Its curves are schematic, with separate within-system scales. Table 2 defines the quantities used throughout the review; Section 9 addresses their estimation rather than redefining them.

## 3. Glucocorticoid Feedback Closure

The hypothalamic–pituitary–adrenocortical (HPA) axis provides the clearest molecular entry point for studying stress termination. Acute challenge recruits corticotropin-releasing hormone and related hypothalamic signals, adrenocorticotropic hormone release, and adrenal glucocorticoid secretion. Glucocorticoids then exert permissive, stimulatory, suppressive, and preparative actions whose direction and timing vary with concentration, receptor occupancy, cell type, and prior state [18,41,42,43]. The post-stressor cortisol trajectory is therefore not passive decay; it reflects continuing secretion, distribution, metabolism, receptor-mediated feedback, and context-sensitive neural control.

Glucocorticoid feedback closure is used here as an operational description of the shift from continuing HPA drive toward feedback-constrained post-peak activity. It is not a directly observed switch, and receptor signaling can continue during declining cortisol. The minimum observational requirement is a series that brackets the individual rise or sustained elevation, peak, and subsequent decline, with repeated prestressor measurements. Dense cortisol sampling is necessary but does not by itself distinguish continued secretion from clearance. Plasma ACTH, a prespecified secretion–elimination model, or a functional GR assay provides additional leverage when a receptor- or secretion-specific interpretation is intended [12,18].

### 3.1. Mineralocorticoid and Glucocorticoid Receptor Dynamics

Mineralocorticoid receptors (MRs) and glucocorticoid receptors (GRs) provide a concentration- and context-dependent architecture for corticosteroid regulation. MRs have high affinity and contribute to appraisal, basal excitability, and response selection, whereas GR engagement becomes increasingly prominent as glucocorticoid concentrations rise and contributes to consolidation, redistribution of resources, and feedback [18,44,45]. The functional result depends not only on receptor abundance but also on receptor localization, co-regulators, chromatin state, and the timing of ligand exposure.

Neural feedback is distributed rather than localized to a single inhibitory center. The hippocampus, medial and lateral prefrontal regions, amygdala-related circuits, hypothalamus, and brainstem influence HPA output through direct and indirect pathways [18,43,46]. Acute stress also changes corticosteroid receptor binding at target genes, including MR–GR heterodimerization in the hippocampus [20]. These observations argue against treating a cortisol decline as a simple readout of one receptor or one brain region.

MR–GR balance is most useful as a mechanistic heuristic, not as a one-dimensional resilience index. Effective transition requires enough early corticosteroid signaling to support coping, allocation of resources, and memory, followed by feedback and cellular recalibration. Prolonged GR resistance may delay closure, whereas premature or excessive suppression could impair functions for which glucocorticoids remain necessary. The relation between receptor signaling and adaptation is therefore expected to be nonlinear and state-dependent.

### 3.2. The FKBP4–FKBP5–SKA2 Regulatory Module

GR function is shaped by an intracellular chaperone complex in which FK506-binding proteins are prominent. FKBP4 generally facilitates dynein-associated receptor transport and productive signaling, whereas FKBP5 reduces GR ligand affinity and nuclear translocation. Glucocorticoid-induced FKBP5 expression then creates an intracellular feedback loop that can limit subsequent receptor sensitivity [21,22]. This property is relevant to stress termination because prior ligand exposure changes the gain of later receptor signaling.

This biology resists one-directional interpretation. Higher FKBP5 is not invariably maladaptive, nor is lower FKBP5 invariably protective; consequences vary with tissue, developmental history, receptor balance, timing, and the magnitude of induction. Preclinical work indicates that MRs can regulate FKBP5 and thereby dampen GR sensitivity [23]. Neuronal evidence also shows that spindle and kinetochore-associated protein 2 (SKA2) can enhance stress-related GR signaling through FKBP4–FKBP5 interactions [24]. The defensible unit of explanation is a context-dependent regulatory module, not an FKBP5 master switch.

This regulatory module motivates a specific, unconfirmed prediction: functional GR sensitivity or a prespecified glucocorticoid-responsive transcript module should explain post-peak cortisol dynamics after initial amplitude and timing are accounted for. A comparison with immediate cardiovascular mobilization tests specificity, rather than assuming it. Many available genetic or expression studies do not contain the dense endocrine measurements needed for this comparison.

### 3.3. Molecular Feedback Closure as a Distributed Program

Feedback closure emerges from a distributed set of processes: continuing CRH and ACTH drive, cortisol delivery and metabolism, MR and GR occupancy, chaperone exchange, receptor trafficking, nuclear translocation, chromatin accessibility, transcriptional feedback, and multisynaptic control from hypothalamic, brainstem, hippocampal, and prefrontal systems [18,19,47]. A fall in peripheral cortisol is consequently a delayed and indirect readout of mechanisms that may compensate for, or obscure, one another.

Time-resolved transcription offers one route toward molecular specificity. Glucocorticoid-responsive targets include FKBP5, TSC22D3/GILZ, DUSP1, and SGK1. Arloth et al. examined genetically moderated transcription following GR activation in human blood and related these responses to brain function and psychiatric risk [25]. This pharmacological GR-response evidence is not a demonstration of transcriptional resolution after a psychosocial stressor. It supports the choice of functional candidate measures, whose recovery-specific meaning requires repeated sampling and independent validation.

**Figure 2:**
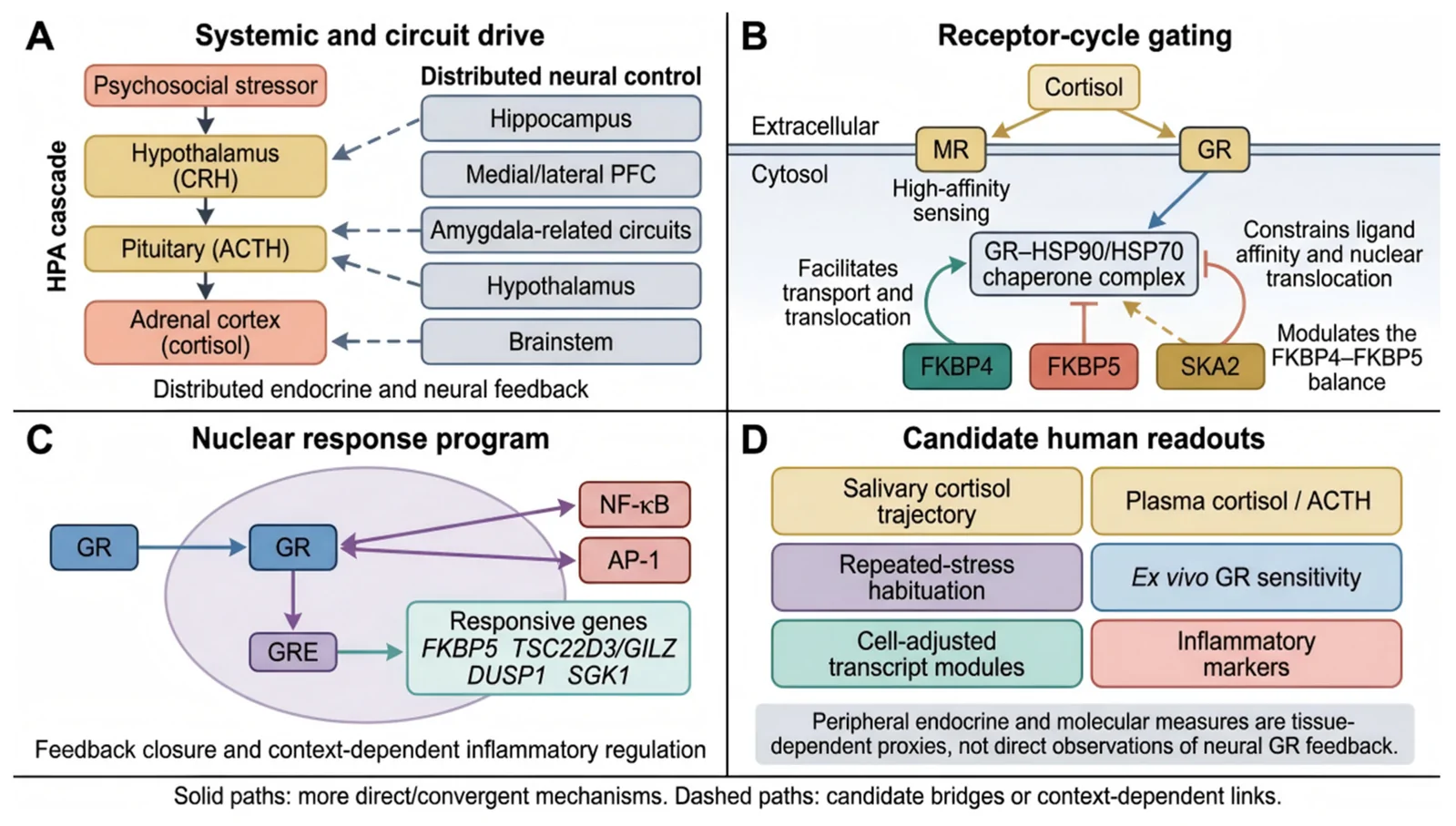
Distributed molecular architecture of glucocorticoid feedback closure. The panels distinguish systemic and circuit drive (**A**), receptor-cycle regulation (**B**), nuclear response programs (**C**), and accessible human assays (**D**). FKBP4, FKBP5 and SKA2 affect GR signaling in context-dependent ways; the diagram is not a binary molecular switch. Solid paths indicate supported component mechanisms and dashed paths indicate indirect or candidate links, not a quantitative evidence grade. Peripheral cortisol or molecular assays cannot directly identify neural GR feedback. ACTH, adrenocorticotropic hormone; AP-1, activator protein 1; CRH, corticotropin-releasing hormone; FKBP, FK506-binding protein; GR, glucocorticoid receptor; GRE, glucocorticoid response element; HPA, hypothalamic–pituitary–adrenocortical; MR, mineralocorticoid receptor; NF-κB, nuclear factor kappa B; PFC, prefrontal cortex; SKA2, spindle and kinetochore-associated protein 2.

Figure 2 separates endocrine drive, receptor trafficking, nuclear regulation, and accessible human assays. Its important constraint is the gap between a supported intracellular mechanism and an indirect peripheral readout. Salivary cortisol, plasma ACTH, ex vivo GR sensitivity, and cell-adjusted transcript modules address different parts of that gap; none alone identifies neural feedback closure.

### 3.4. Genetic and Epigenetic Modifiers: Evidence and Limits

FKBP5 variation and childhood trauma have been linked to allele-specific demethylation and enduring differences in stress-related regulation [48]. Earlier studies also reported associations between FKBP5 polymorphisms and cortisol recovery after psychosocial stress, between FKBP5 or CRHR1 variation and acute cortisol responses, and between NR3C1 methylation and HPA-axis regulation in adolescents [49,50,51]. These findings provide biological plausibility for moderated feedback gain, but they do not establish a general genetic architecture of stress termination.

These limitations constrain inference, not simply precision. Candidate-gene studies are often small relative to the expected effects on behavioral and endocrine phenotypes. Methylation measured in blood or saliva is an imperfect proxy for receptor regulation in specific neural or immune cell populations; cell composition, medication, smoking, age, adversity, and current stress can confound associations; and developmental stage can alter the meaning of the same mark. A preregistered 2026 systematic review and meta-analysis found heterogeneous, low-certainty evidence overall, with the most consistent NR3C1 methylation association confined to infant cortisol responses rather than generalized across the life span [52]. In the PSTM, genetic and epigenetic variables therefore modify gain and plasticity; they do not determine a fixed transition type.

Acute stress may itself produce detectable epigenomic change. A recent within-person genome-wide study reported both hypermethylation and hypomethylation in salivary DNA after acute stress, while underscoring the difficulties posed by tissue composition, sampling time, and functional interpretation [53]. Together with evidence linking stress- and age-related FKBP5 upregulation to NF-κB-driven inflammation [54] and childhood abuse to altered glucocorticoid receptor regulation in human hippocampus [55], these results support biological embedding without validating a single peripheral epigenetic marker of recovery. Future studies should favor genome-wide or transcriptomic designs, repeated sampling, cell-composition adjustment, preregistration, and independent replication over isolated candidate associations [56].

Accordingly, an epigenetic difference in blood or saliva is treated as a potential correlate or modifier of stress regulation. Without tissue-relevant functional evidence and temporal linkage, it is not an indicator that a neural termination mechanism is engaged, impaired, or responsible for the observed trajectory. Peripheral association, mechanistic mediation, and central localization are separate claims.

### 3.5. From Molecular Feedback to Human Cortisol Trajectories

Salivary cortisol indexes peripheral free cortisol with sampling and transport constraints; it does not directly measure instantaneous central drive. Circadian phase, sampling compliance, contamination, assay characteristics, recent intake, and oral conditions can affect the observed trajectory [7,8,9]. The individual peak can occur after the task ends. Defining recovery from a fixed first post-task sample therefore mixes continued rise in some participants with post-peak decline in others.

The model-based comparison by Miller et al. demonstrates that estimated reactivity and recovery depend on curve assumptions and sampling design [12]. AUC remains useful for exposure but does not isolate feedback [11]. Section 9.1 specifies a literature-informed sampling floor and Section 9.2 addresses late peaks and nonresponse. These recommendations target observable dynamics; stronger mechanistic claims require the additional measurements described above.

High, low, and flat cortisol trajectories cannot be interpreted outside their context. Chronic stress and psychiatric disorders have been associated with both increased and attenuated responses, depending on chronicity, diagnosis, medication, sex, and design [57]. Early adversity is associated with blunted cardiovascular and cortisol reactivity at the meta-analytic level, but attenuation is not synonymous with resilience [58]. The PSTM therefore locates risk in the joint pattern of mobilization, peak timing, feedback-related decline, and cross-system organization, not in response amplitude alone.

## 4. Vagal Re-Engagement and Peripheral Autonomic Transition

Vagal re-engagement denotes inferred post-stressor cardiac dynamics compatible with increasing parasympathetic influence; it is not a direct recording of vagal nerve activity. A fall in heart rate or blood pressure can also reflect sympathetic withdrawal, altered breathing, baroreflex changes, or reduced movement. The construct therefore requires convergent, respiration-aware interpretation rather than any isolated increase in HRV.

High-frequency HRV and RMSSD are commonly used indices of vmHRV, but their interpretation depends on recording duration, stationarity, posture, breathing, ectopy, preprocessing, and mean heart period [27,59,60]. They do not identify central vagal firing or separate all autonomic mechanisms. Respiratory measurement is particularly important during the transition from speaking and mental arithmetic to seated rest.

### 4.1. Sympathetic Withdrawal Is Not Parasympathetic Return

Autonomic branches can change independently. Sympathetic withdrawal may lower heart rate and vascular drive while parasympathetic activity remains suppressed; conversely, vagal influence can increase while sympathetic activity is still elevated. A single cardiac measure cannot identify these alternatives. Designs that combine ECG, impedance cardiography, blood pressure, electrodermal activity, respiration, and—where feasible—pre-ejection period provide a more interpretable decomposition of post-stressor change.

Neurovisceral integration models place prefrontal, cingulate, insular, amygdalar, and brainstem regions within a distributed network that supports flexible autonomic regulation [61,62,63]. In the PSTM, vagal re-engagement is both a candidate effector and a source of feedback: updated context, interoceptive evidence, and central control are translated into cardiovascular stabilization, while changing bodily signals update the central state. The timing of vmHRV recovery should therefore covary with neural and cognitive change under some conditions, but the model does not require a uniform association across stressors or individuals.

### 4.2. What Current Recovery Studies Establish

Evidence for post-stressor vagal dynamics is suggestive but methodologically heterogeneous. In a small study of healthy men, lower resting RMSSD was associated with poorer recovery of diastolic blood pressure, cortisol, and tumor necrosis factor-α after acute stress [28]. Other work links anticipatory HRV change to the subsequent cortisol response, consistent with coordination between autonomic preparation and endocrine mobilization [64]. Such findings support cross-system coupling, but their sample sizes and observational nature preclude a universal causal interpretation.

A recent scoping review found substantial variation in stress paradigms, recovery-window definitions, body position, HRV metrics, respiration control, and analytic methods [29]. Broader reviews likewise indicate that HRV is sensitive to psychological stress but is not process pure [65,66]. A five-minute segment beginning immediately after a speech may contain movement, speech-related respiratory carryover, and rapid nonstationarity; a segment beginning later may miss the fastest transition. Neither segment can be labeled the recovery period without specifying what process it is intended to capture.

Current evidence supports three bounded conclusions. Autonomic recovery contains information not captured by peak heart rate alone; the meaning of vmHRV change depends on respiration and baseline state; and recovery is better represented by a trajectory or a sequence of windows than by one arbitrary difference score. The evidence does not yet justify a universal healthy recovery slope, a stand-alone vmHRV resilience biomarker, or a central vagal mechanism inferred from a brief peripheral recording.

### 4.3. Perseverative Cognition and Vagal Re-Engagement

The perseverative cognition hypothesis proposes that worry, rumination, and anticipatory processing prolong stress-related physiology beyond direct exposure to a stressor [35]. Meta-analytic evidence links perseverative cognition to modest changes across cardiovascular, endocrine, and autonomic outcomes [36]. In the post-stressor period, the relevant question is not simply whether a person reports rumination, but whether the internal representation of current threat remains active and predicts delayed autonomic transition.

Studies of adolescent depression and female samples have associated rumination with impaired or prolonged cortisol recovery [67,68]. A 2026 series of meta-analyses reported associations between perseverative cognition and lower vmHRV across laboratory contexts, with relatively small recovery-period effects and greater support in within-person than between-person contrasts [37]. This pattern is compatible with a dynamic effect of momentary cognition, but cautions against interpreting a person-level rumination score as a universal cause of autonomic dysregulation.

A post-offset manipulation can test whether reducing current-threat appraisal precedes changes in respiration-aware vmHRV and later cortisol. Assessment is itself a possible perturbation: repeatedly asking about errors, rumination, or evaluation may sustain the process under study. Brief, neutrally worded probes should separate current threat from repetitive thought without requiring event rehearsal. Probe timing and duration should be logged, and a pilot or randomized sparse-probe/no-probe comparison should test assessment reactivity. Temporal analyses should treat probe occasions as potential inputs rather than assume that measurement is inert.

### 4.4. Respiratory and Biofeedback Interventions as Mechanistic Probes

Slow breathing and HRV biofeedback can amplify cardiorespiratory oscillation and baroreflex engagement, and meta-analyses support benefits for stress- and anxiety-related outcomes [69,70,71]. These methods are informative for the PSTM because they can be delivered after t_0_, perturbing an early transition process without changing the stressor. Interpretation still requires restraint: breathing rate and tidal volume can mechanically increase HRV, and clinical improvement does not show that vagal re-engagement mediated the effect.

Respiratory handling should follow the estimand [27,60]. For spontaneous recovery, retain spontaneous breathing, continuously record respiratory rate and a calibrated or clearly labeled relative amplitude measure, and standardize speech and posture. Paced breathing is useful in a separate standardized comparison when respiratory variation is intentionally constrained; it should not replace natural recovery in the primary observation. In a slow-breathing or biofeedback trial, pacing is an active manipulation, so respiration is partly a mediator rather than merely a nuisance covariate.

Report raw cardiac dynamics together with prespecified respiration-adjusted sensitivity analyses, separating within-person respiratory change from between-person differences. Adjustment cannot make HRV a direct neural measure and may remove part of the treatment pathway. Slow breathing can move respiratory oscillation outside the conventional high-frequency band; RMSSD, the measured breathing frequency, and the full spectral context should then be reported rather than relying on high-frequency power or LF/HF alone. Respiratory-phase gating is appropriate for phase-specific evoked analyses, not a general correction for whole-window HRV recovery. Short-window estimates require validation against longer, approximately stationary segments within the intended protocol [27,59,60].

## 5. Frontolimbic Context Updating and Cognitive Disengagement

Frontolimbic context updating is a multilevel construct linking recognition that a stressful event has ended to distributed prefrontal, hippocampal, limbic, salience, and interoceptive dynamics. It does not designate a dedicated termination circuit. After social evaluation, anticipated judgment, uncertainty about consequences, or interpretation of residual arousal can maintain an internal representation of current threat. The hypothesis is that updating this representation can change subsequent regulation; it is not that cognitive recognition is necessary for every physiological decline.

Context updating should not be equated with positive reappraisal or immediate emotional relief. It concerns the inferred state of the environment and the action policy appropriate to that state. Recognition of event completion, revised controllability, cessation of performance monitoring, and reallocation of attention can all contribute. Negative memory and delayed endocrine activity may persist even after the context has been correctly updated.

### 5.1. Stress-Related Network Reconfiguration

Acute stress recruits noradrenergic and glucocorticoid mechanisms that reorganize large-scale brain networks toward salience detection and rapid action [72,73]. Immediate post-stress studies report altered amygdala connectivity with hippocampal and cortical midline regions [74,75,76]. These changes are consistent with continued prioritization of salient and self-relevant information, but their adaptive value depends on timing and context. The configuration that supports threat detection during challenge may become costly if it persists after threat has ended.

The triple-network framework offers a useful vocabulary for this process: salience-network engagement, executive-control allocation, and default-mode self-referential processing can be rebalanced during and after stress [77]. Recovery, however, is unlikely to be captured by every static connection returning to its prestress value. Network-state occupancy, dwell time, transition probability, integration, and metastability may be more informative. A recent multiphase fMRI study documented rapid and partly reversible large-scale reconfiguration from stress induction into the post-stress period [30], illustrating the value of phase-resolved state analysis.

Convergent evidence remains preliminary. A longitudinal study of military personnel identified post-stress and recovery-related changes in whole-brain network dynamics, with selected features associated with later vulnerability to real-life chronic stress [31]. Increased dorsal-attention-network centrality has also been reported during recovery [32]. Samples are modest, recovery intervals differ, and state solutions depend on preprocessing and model choice. The immediate goal should therefore be replication of preregistered transition parameters across tasks and cohorts, not declaration of a universal neural signature of successful recovery.

### 5.2. Prefrontal–Hippocampal Regulation and Event Closure

Prefrontal and hippocampal systems contribute context, episodic structure, control, and valuation, and influence HPA output through multisynaptic pathways [46,78,79]. Stress-related prefrontal disruption has been observed under sustained real-life stress and acute laboratory challenge, which should not be treated as the same exposure [80,81]. These findings provide a substrate for context-updating hypotheses but do not demonstrate a specific event-closure mechanism.

The cognitive consequences of acute stress are heterogeneous rather than uniformly impairing. Meta-analyses show that executive and episodic-memory effects depend on timing, task, cortisol, and memory phase [82,83]. Stress can also undermine deliberate emotion regulation and shift goal-directed choice [84,85]. The implication for recovery is not that cognition must normalize before physiology can change, but that the capacity to revise the current context may be constrained precisely when event closure is required.

Phase-resolved connectivity findings link this circuitry more directly to HPA dynamics. Limbic connectivity during stress induction and post-stress rest has shown different associations with cortisol reactivity and recovery; post-stress prefrontal coupling with basolateral amygdala or hippocampus appears particularly relevant [33]. Objective sleep efficiency has also been associated with cortisol recovery and phase-dependent dorsolateral prefrontal–hippocampal coupling, although the reported mediation remains correlational [34]. Chronic stress may further alter the relation between acute cortical–limbic dynamics and depressive symptoms [86]. Together, these studies support phase specificity while leaving direction and generalizability unresolved.

### 5.3. Controllability, Predictability, and Perseverative Cognition

Social-evaluative threat is potent partly because performance is visible, outcomes are uncertain, and control is constrained [6]. At t_0_, objective task demands cease, but perceived uncontrollability or uncertainty may persist. A person may believe that evaluation continues outside the room, that consequences remain unknown, or that further action is required. Such beliefs can maintain salience and prevent disengagement even in the absence of new external threat.

Perseverative cognition is one expression of incomplete context updating, but it is not the whole process. Rumination can revisit a completed event while correctly recognizing that it is over; conversely, a person may report little verbal rumination yet remain in a nonverbal state of anticipatory vigilance. Repeated assessment should therefore distinguish event completion, current threat, controllability, uncertainty, self-evaluation, and repetitive thought. Collapsing them into a single negative-affect score makes the mechanism difficult to test.

Induced rumination has been associated with delayed cortisol recovery, and one EEG study reported moderation by scalp connectivity measures described as prefrontal–limbic [87]. Scalp connectivity is not direct recording of a deep limbic pathway and cannot identify the direction of a neural mediator. A post-offset information or rumination manipulation, with respiratory measurement and temporally separated neural and endocrine outcomes, is required to test the proposed sequence.

### 5.4. Interoceptive Inference During Recovery

Post-stressor bodily signals are ambiguous. Palpitations, warmth, trembling, breathlessness, and residual arousal can be interpreted as normal physiological lag, evidence of continuing danger, evidence of poor coping, or signs of illness. Interoceptive brain systems integrate visceral signals with prior beliefs and contextual predictions [88,89,90]. The interpretation of residual arousal may therefore influence whether the stressor is represented as complete.

The model makes no assumption that more accurate detection of bodily signals necessarily accelerates recovery. Interoceptive attention may reveal declining arousal, but it can also intensify monitoring and catastrophic interpretation. Precision, appraisal, and context are the critical variables. One testable prediction is that maladaptive beliefs about residual arousal will prolong current-threat representation and autonomic instability after the magnitude of the bodily signal itself has been controlled.

This proposition creates a bridge between molecular and psychological levels without reducing one to the other. Cortisol and autonomic signals change the interoceptive evidence available to the brain; beliefs and attention alter the inferred meaning and behavioral response to those signals. Bidirectional models are therefore more plausible than a one-way account in which either physiology or cognition simply drives the other.

### 5.5. Sleep as a State Modifier of Context Updating

Sleep affects prefrontal regulation, amygdala responsivity, autonomic function, inflammation, and HPA activity. Poor habitual sleep efficiency has been associated with increased cardiovascular and cortisol reactivity, and experimental sleep deprivation can potentiate HPA responses [91,92]. Day-to-day sleep variation may add information beyond average sleep, although conclusions depend on how both sleep and cortisol trajectories are parameterized [93].

Sleep is particularly relevant to post-stressor transition because it may alter the neural resources available for context updating and the gain of autonomic or endocrine feedback. Naturally occurring sleep loss has been associated with reduced post-stress amygdala resting-state connectivity [94], and recent work links objective sleep efficiency to cortisol recovery and dynamic prefrontal–hippocampal regulation [34]. The evidence remains insufficient to conclude that sleep selectively affects recovery rather than reactivity. Repeated within-person manipulation or naturalistic monitoring is required to test that specificity.

### 5.6. Causal Evidence from Prefrontal Neuromodulation

Neural stimulation provides a direct test of prefrontal involvement when delivered after the stressor. In one study, repetitive transcranial magnetic stimulation over the left dorsolateral prefrontal cortex was followed by faster cortisol recovery [95]. Sham-controlled theta-burst findings suggest that post-stress effects may depend on individual traits and protocol features [96]. These studies provide useful proof of concept, but they do not establish a universal target or identify the intervening neural–autonomic–endocrine pathway.

Future trials need a state assessment immediately before stimulation, credible sensory controls, and temporally ordered neural, autonomic, endocrine, and subjective outcomes. A sequence in which stimulation first changes current-threat inference or network state, followed by vmHRV and then cortisol, would support one version of the PSTM. A cortisol effect without measurable neural–cognitive or autonomic intermediates would require another account. Null effects among individuals who already transition efficiently would also be compatible with state-dependent prefrontal involvement.

### 5.7. Why These Regulatory Functions, and Where Other Mediators Enter

The three organizing functions were selected because they address distinguishable constraints on the transition: whether threat remains current, how cardiac autonomic influence changes, and how continuing endocrine output is regulated. Each has a mechanistic literature, an observable human correlate, and at least one candidate perturbation. These are functional levels, not three mutually exclusive molecular systems. The selection is parsimonious and provisional; it does not establish that these functions are uniquely necessary or sufficient, or that other mediators are less important.

Catecholaminergic and endocannabinoid signaling operate across this architecture. Noradrenergic signaling supports stress-related network reconfiguration and prefrontal control [72,78,79]; sympathetic catecholaminergic withdrawal also contributes to cardiovascular change without being equivalent to cardiac vagal return [43]. In rodents, local prefrontal CB_1_-receptor antagonism prolonged post-stress corticosterone secretion, and glucocorticoid-dependent endocannabinoid signaling provided a link between receptor activation and cortical regulation [97]. Human stress-induced changes in circulating anandamide support peripheral responsivity, not a direct measure of central CB_1_-mediated termination [98]. Endocannabinoids are therefore a candidate mechanism linking neural and endocrine regulation, not an excluded fourth competitor.

Oxytocin and arginine vasopressin (AVP) provide further, non-equivalent routes. Endogenous oxytocin responses have been associated with stronger cortisol reactivity and faster vagal recovery [99]. Intranasal oxytocin combined with social support has reduced cortisol responses, but administration before stress tests buffering as well as subsequent recovery and does not isolate a post-offset effect [100]. Central–peripheral oxytocin correspondence varies with experimental context [101]. AVP can potentiate pituitary ACTH drive; changes in copeptin during psychosocial stress provide an accessible peripheral correlate of the vasopressin system, not proof of central termination signaling [18,102]. These mediators may alter shared inputs, response gain, or feedback across the three functions. Their inclusion does not imply that increasing or decreasing any one of them universally improves recovery.

## 6. Delayed Immune and Transcriptional Resolution

Repeated human sampling indicates a broad temporal tendency rather than a fixed autonomic–cortisol–cytokine chain. Cardiovascular changes can occur during and immediately after challenge, salivary cortisol often peaks in the ensuing tens of minutes, and circulating IL-6 changes commonly become clearer later. Meta-analyses support delayed inflammatory responses, especially IL-6, but timing depends on the marker, stressor, assay, and whether time is measured from stress onset or offset [103,104]. A late rise is not evidence that immune resolution has already been measured.

Acute immune change is not intrinsically harmful. It can redistribute cells, support tissue surveillance, and coordinate repair, whereas repeated or chronically unresolved activation may become costly [105]. The relevant questions are whether the response is proportionate, temporally bounded, and appropriately regulated by endocrine and autonomic systems. Social threat also engages neural–immune pathways implicated in depression and related conditions, but acute laboratory dynamics should not be equated with chronic disease mechanisms [106].

In 21 men exposed to three TSST sessions one week apart, von Känel et al. sampled plasma IL-6 at rest, immediately after stress, and 45 and 105 min later. IL-6 increased at the later observations without clear habituation, whereas cortisol and systolic-pressure responses habituated across sessions [38]. McInnis et al. measured IL-6 before, 30 min after, and 120 min after repeated psychosocial stress on consecutive days, alongside more frequent salivary cortisol samples. The two systems showed different adaptation patterns, including IL-6 sensitization in contrast to cortisol habituation [39]. These studies demonstrate dissociable time courses, not that one pattern is inherently maladaptive.

Repeated-stress gene-expression work further distinguishes circulating mediators from cellular responses: pro- and anti-inflammatory transcripts can respond and habituate differently from plasma cytokines [26]. Together with stress-related NF-κB and cytokine measurements [40], this supports sampling leukocyte composition, transcription, and soluble proteins separately. A design ending at 90–120 min may capture delayed inflammatory activation but not its descending limb; demonstrating resolution may require a later observation chosen from pilot kinetics. Peripheral transcript changes also need not occur after the cytokine peak. Immune timing is therefore a system-specific hypothesis, not a universal serial endpoint.

### 6.1. GR-Inflammatory Crosstalk

Glucocorticoid signaling interacts with inflammatory transcription through multiple mechanisms, including GRE-dependent regulation and protein–protein interactions with nuclear factor-kappaB and activator protein-1 [107]. Chronic insufficient glucocorticoid signaling or reduced sensitivity can permit sustained inflammatory activity even when circulating cortisol is elevated [108]. In healthy men, acute psychosocial stress has been shown to increase NF-κB binding activity in association with subsequent pro-inflammatory gene expression, demonstrating that transcriptional events can unfold after the overt challenge [40]. Neural and innate immune systems also regulate one another reciprocally through autonomic, endocrine, and cytokine pathways [109].

Delayed immune and transcriptional activity is therefore an informative but nonspecific layer of the PSTM. A single cytokine cannot serve as a direct measure of GR closure. Cytokine concentrations reflect production, clearance, leukocyte redistribution, infection, adiposity, reproductive and circadian factors, and assay variation; peripheral blood also samples only part of a tissue-specific response. Mechanistic studies should pair time-resolved trajectories with cell-composition information, glucocorticoid-responsive transcript modules, or functional sensitivity assays where feasible.

### 6.2. From Acute Resolution to Repeated Biological Embedding

The translational importance of incomplete transition is likely to lie in repetition. If an episode ends with persistent threat representation, delayed vagal re-engagement, inefficient GR feedback, or unstable immune resolution, the next challenge may begin before the preceding state has re-equilibrated. Recurrent overlap could alter baseline state, receptor sensitivity, sleep, and behavior. This offers a plausible route from acute dynamics to allostatic load without classifying one delayed response as pathological.

Prospective designs are indispensable. A transition parameter estimated in an initial session should predict habituation or sensitization during a later standardized stressor, recovery after naturally occurring stress, or subsequent symptoms and function. Cross-sectional associations with current distress cannot establish direction because symptoms may shape the laboratory response, the response may influence symptoms, or both may reflect prior exposure. Clinical relevance requires reliability, prospective validity, and modifiability.

The evidence base is uneven. Receptor biology and HPA feedback have strong mechanistic foundations; post-stressor vmHRV has moderate but heterogeneous support; phase-specific neural and cognitive recovery is an emerging literature; and the prospective validity of cross-system lags remains largely untested. Table 1 separates established mechanisms from variable human evidence and from propositions that are specific to the PSTM.

## 7. The Post-Stressor Transition Model

The PSTM links the functions developed in Section 3, Section 4, Section 5 and Section 6 to explicit tests of post-offset regulation. Its contribution is operational rather than the discovery that recovery matters. Allostatic accounts already explain context-sensitive resource allocation and cumulative cost [1,2]; neurovisceral integration already links central regulation with autonomic flexibility [61,62]; and perseverative-cognition accounts already explain activation beyond direct exposure [35]. Peak-adjusted models separate endocrine response components [12,13], while multi-trajectory models characterize coordinated heterogeneity [14,15,16,17]. None should be treated as a null theory that ignores recovery.

The proposed extension is to preserve offset and individual extrema simultaneously, define adaptation using outcomes outside the fitted trajectory, and compare mechanistically constrained pathways under interventions applied after offset. A slower cortisol tail can then be tested as residual secretion, altered feedback, a shared input, or a response to sustained threat representation. Merely naming a profile or fitting a better curve does not establish a new mechanism. Supplementary Table S1 sets out the overlap and the additional test required relative to each preceding framework.

**Figure 3:**
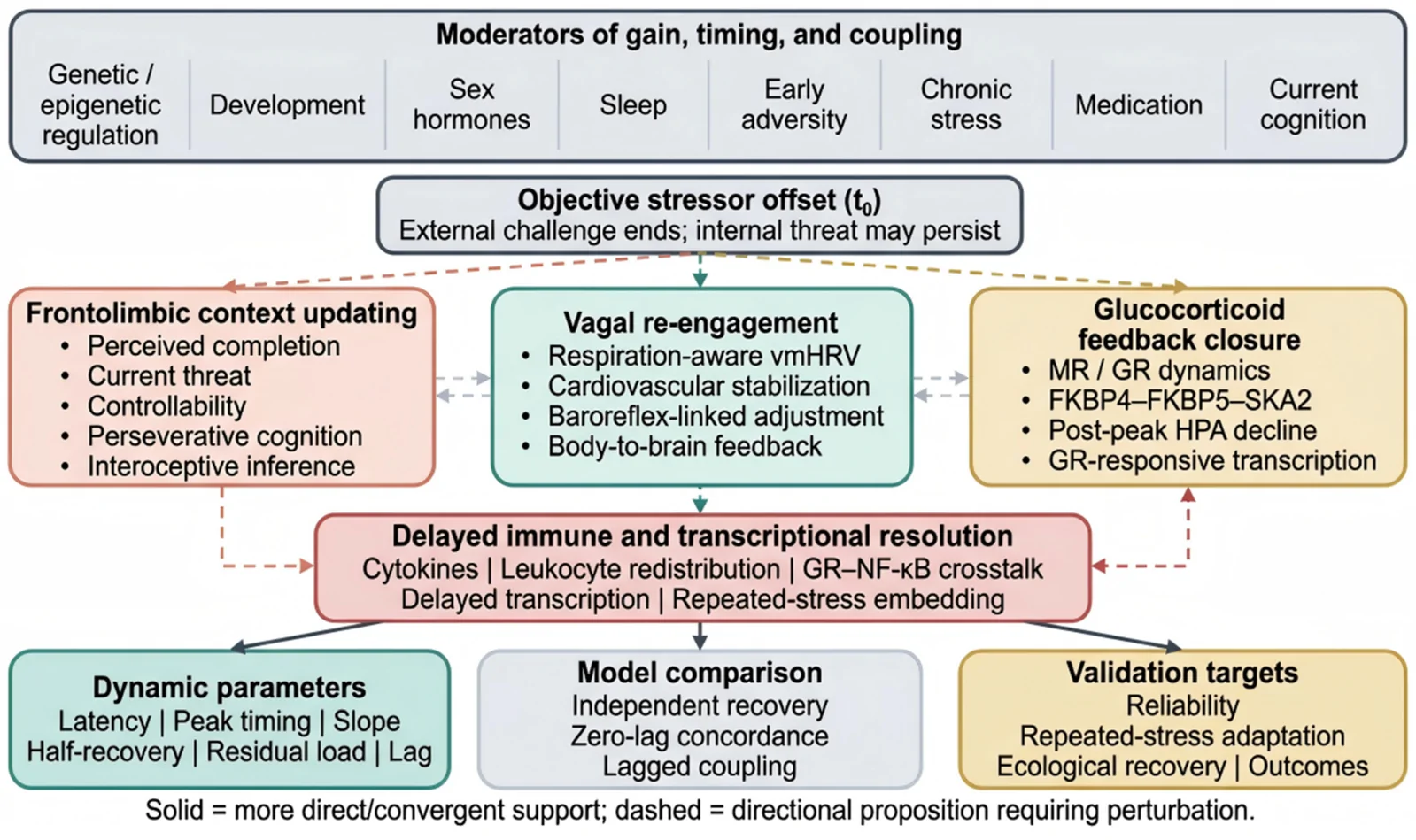
The Post-Stressor Transition Model (PSTM). Context updating, inferred cardiac vagal re-engagement, and glucocorticoid feedback closure organize candidate post-offset regulatory functions; they are not an exhaustive list of molecular systems. Their central links are hypotheses for comparison with shared-input and independent-recovery accounts. The immune/transcriptional layer can influence and be influenced by these processes. Moderator effects are tested on gain, reference level, timing, return rate, or coupling rather than assumed to define fixed types. The lower row specifies the measurements and validation needed before functional or clinical interpretation. GR, glucocorticoid receptor; HPA, hypothalamic–pituitary–adrenocortical; NF-κB, nuclear factor kappa B; vmHRV, vagally mediated heart rate variability.

Figure 3 assembles the three functions and the delayed immune layer. The central cross-system paths are candidate couplings, not an established sequence. The figure’s lower row is essential: each proposed relation must survive measurement checks, competing-model comparison, and independent validation. The additional mediators in Section 5.7 can act within or across these paths without changing the three-function organization.

A useful starting point is a state-space representation of deviations from a person- and session-specific reference. With z_s_(t) = x_s_(t) − b_s_, one candidate dynamic equation is:

dz_s_(t)/dt = −k_s_z_s_(t) + ∑_j≠s_ a_js_z_j_(t − τ_js_) + γ_s_C(t) + β_s_U(t) + η_s_(t). (1)

Here, k_s_ describes intrinsic return, a_js_ a candidate cross-system influence, and τ_js_ an explicit delay when the sampling design can identify it. C(t) represents shared, measured or latent input; U(t) represents an assigned intervention. The process term η_s_(t) is distinct from observation error. Observed y_s_(t_i_) is linked to the latent state through a system-specific measurement function and, where justified, a transport or averaging filter. Centering the coupling terms avoids a nonzero equilibrium generated solely by other systems’ baselines. This equation is a candidate model family, not a fitted or validated PSTM parameterization.

Independent, shared-input, coupled, and explicit-delay variants must use comparable observations and measurement assumptions. In particular, shared input plus different sensor kinetics can generate apparent lags without directional coupling. Section 9.3 specifies statistical comparisons; only parameters distinguishable under those comparisons should receive a mechanistic interpretation.

### 7.1. Eight Falsifiable Propositions

Table 3 defines eight confirmatory tests with prespecified contrasts and time windows. The windows are planning proposals for adult social-evaluative stress, not established biological cut-offs. Each study must define a smallest effect size of interest (SESOI), the scale of its effect, and its primary endpoint before examining outcomes. For illustration, a protocol might choose |d| = 0.20 for a standardized intervention contrast or ΔR^2^ = 0.02 for incremental prospective prediction. These are transparent design conventions, not effects estimated by this review, universal meaningfulness thresholds, or claims of adequate power.

Disconfirmation requires an informative test. A nonsignificant result with wide uncertainty is inconclusive, whereas a confidence interval excluding the prespecified meaningful effect, equivalence within justified bounds, a reproducible effect in the opposite direction, or superior prediction by a competing model weakens the specified claim [110]. For a two-sided equivalence test at α = 0.05, a 90% confidence interval within the prespecified bounds supports equivalence; for a directional minimum-benefit claim, the corresponding one-sided bound is appropriate. A reliable central prediction cannot be rescued after an informative null by changing the outcome, lag, subgroup, or threshold. Broader observation of different system kinetics may remain valid even when a particular PSTM path fails.

### 7.2. Candidate Dynamic Profiles and the Risk of Premature Classification

**Figure 4:**
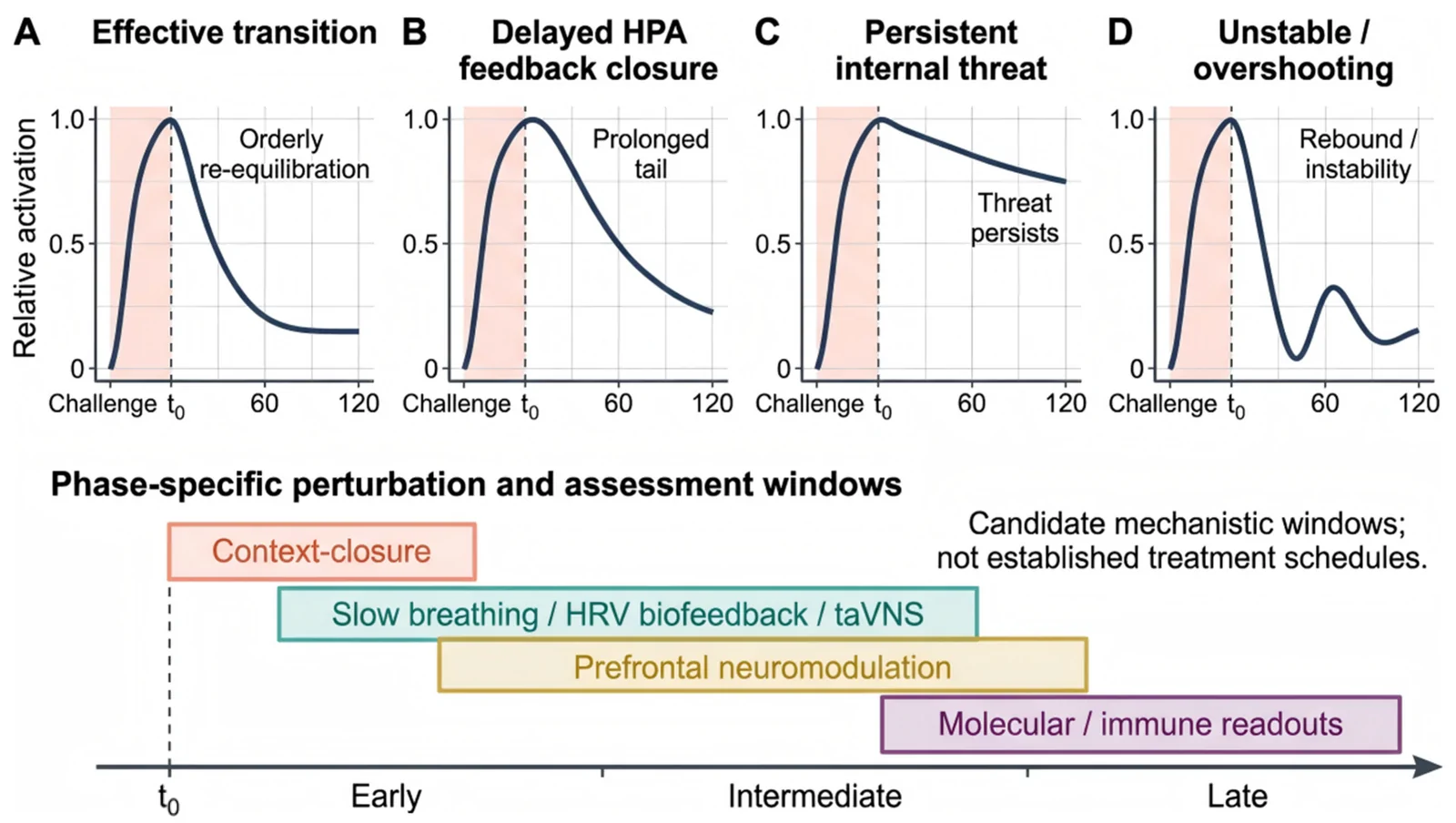
Hypothesized post-stressor profiles and candidate perturbation or assessment windows. Schematic trajectories illustrate orderly re-equilibration, prolonged endocrine decline, continuing threat representation, and unstable change. The displayed relative activation and times are illustrative, not empirical cut-offs; similar appearance does not establish a common mechanism. “Effective” denotes a hypothetical outcome to be tested, not a classification from curve shape. The lower band distinguishes early interventions from later molecular or immune observation. A post-offset intervention can still change an unobserved cortisol peak. HPA, hypothalamic–pituitary–adrenocortical; HRV, heart rate variability; taVNS, transcutaneous auricular vagus nerve stimulation.

Dense sampling may reveal recurring profiles: strong reactivity followed by efficient transition; autonomic improvement with delayed HPA closure; persistent current-threat representation accompanied by attenuated vagal re-engagement; or unstable, overshooting dynamics. Figure 4 illustrates these hypotheses and candidate windows for experimental perturbation. The profiles are not diagnostic classes.

Mixture models can reveal heterogeneity, but apparent classes may arise from skewed continuous distributions, sparse sampling, measurement error, or arbitrary model choice. Multitrajectory studies demonstrate the potential of this approach [14,17], yet classes should not be named as biological subtypes without stability under resampling, replication in an independent cohort, and external validation. Continuous parameters and theory-constrained models should remain primary unless evidence for discreteness is compelling.

### 7.3. A Decision Framework for Resolution and Adaptation

Classification begins with data adequacy. Was the challenge experienced as ended, is the reference state appropriate, and were the relevant extremum and later observations captured with acceptable signal quality? If not, the result is indeterminate. Next, report system-specific resolution continuously: post-extremum slope, residual deviation, and stability within an individually estimated reference or prediction band. A study may prespecify a 95% matched no-stress prediction band and a dwell criterion, such as two consecutive scheduled observations, as an operational convention. The band must account for time of day, posture, breathing, assay uncertainty, and serial dependence; it is not a clinical normal range.

Physiological resolution is then separated from adaptation. A transition is described as provisionally adaptive only when its dynamics are associated with an independently specified favorable outcome, such as preserved subsequent performance, reduced event-related interference, or better prospective recovery, without evidence of a relevant trade-off. Persistent activation may be appropriate if threat remains. A delayed cortisol peak or absent cortisol response cannot establish maladaptation. Until validated outcome-linked thresholds exist, the defensible labels are resolved within the observed window, unresolved within that window, or indeterminate, accompanied by continuous estimates. Supplementary Methods provides a decision checklist rather than a diagnostic algorithm.

## 8. Moderators of the Post-Stressor Transition

Age, hormonal state, circadian phase, sleep, medication, prior exposure, and current symptoms can affect different parameters rather than impose one uniformly healthy trajectory. The PSTM locates these effects on the reference level b_s_, response gain, extremum timing, return rate k_s_, or coupling parameters a_js_. Moderator analyses should identify the target parameter in advance and distinguish within-person state changes from between-person differences.

### 8.1. Development and the Limits of Adult Generalization

The quantitative examples and sampling proposals in this review are adult-focused because the principal evidence and perturbation paradigms concern adult psychosocial stress. Development is not assumed to leave the model’s parameters unchanged. Studies spanning childhood, young adulthood, and older age show measure- and sex-dependent HPA differences [111]. Pubertal research links developmental transition to changes in HPA activity [112], and adolescent work demonstrates coordination, but not identity, between autonomic and endocrine responses [15]. These findings justify age-sensitive models; they do not supply a universal childhood-to-aging recovery slope.

Chronological age alone may be inadequate: pubertal stage, caregiver presence and task meaning matter in younger samples, while comorbidity, medication and reproductive aging complicate older-adult inference. Adult response thresholds, breathing bands, sampling windows, or event-closure manipulations should therefore not be transferred without validation. Development may change both b_s_ and the response/return parameters; the direction of a specific recovery effect remains a hypothesis when only reactivity evidence is available.

### 8.2. Sex and Reproductive Hormonal State

Sex-related and reproductive-hormone effects need to be separated from a universal female-versus-male recovery rule. Laboratory studies and reviews show that salivary free cortisol, plasma total cortisol, and ACTH can differ with sex, menstrual phase, and oral contraceptive use [113,114,115]. Hormone-related binding-protein effects can also change correspondence between total and free measures. These findings support moderator effects on baseline, gain, and peak timing; they do not establish a single direction for the post-peak return rate across women or menstrual phases.

A falsifiable model-level test compares a gain-only account with a model allowing hormonal state to modify k_s_ or cross-system coupling, after measurement and response amplitude are accounted for. Repeated within-person phase comparisons are preferable for that question where feasible; a credible null can support the simpler gain-only account. Minimum reporting includes sex and gender variables as actually collected, menstrual status and phase-estimation method, cycle regularity and relevant dates, hormonal contraceptive formulation and regimen, menopausal status, and hormonal therapy type and timing. Pregnancy or lactation and circulating hormone assays should be reported when relevant. These descriptors remain necessary even when the sample cannot support formal interaction tests [113,114,115].

### 8.3. Prior Exposure and Current Clinical State

Early adversity and chronic stress may produce either sensitized or attenuated response patterns. Meta-analytic evidence links adverse childhood experiences to blunted cardiovascular and cortisol reactivity [58], while chronic stress has been associated with heterogeneous HPA patterns [116]. A low response can therefore reflect efficient calibration, anticipatory elevation, reduced capacity, habituation, or chronic dysregulation. Transition measures may help distinguish these alternatives, but only when baseline and repeated exposure are measured.

Psychiatric symptoms similarly should not be expected to map onto one abnormal trajectory. Cortisol reactivity differs across disorders and designs, with substantial heterogeneity [57]. Depression may be associated with rumination-related delayed recovery in some contexts, while anxiety may be associated with persistent threat representation or altered autonomic regulation. Dimensional moderators—perseverative cognition, anhedonia, uncertainty intolerance, sleep disturbance, and interoceptive beliefs—may be more mechanistically informative than diagnostic group alone.

These moderators are hypotheses about identifiable parameters, not an invitation to search every subgroup. Underpowered interactions should be reported as uncertain; failure to detect them does not establish invariance. The analysis should retain the same outcome definitions across groups and test whether the measurement model itself changes before attributing differences to biology.

## 9. Methodological and Computational Priorities

A transition study must record the end of each stressor component, including evaluation, recording, feedback, and uncertainty. Debriefing is a potential intervention. Standardization of instructions, posture, speech, movement, intake, and device handling is therefore part of identification, not merely procedural detail.

**Figure 5:**
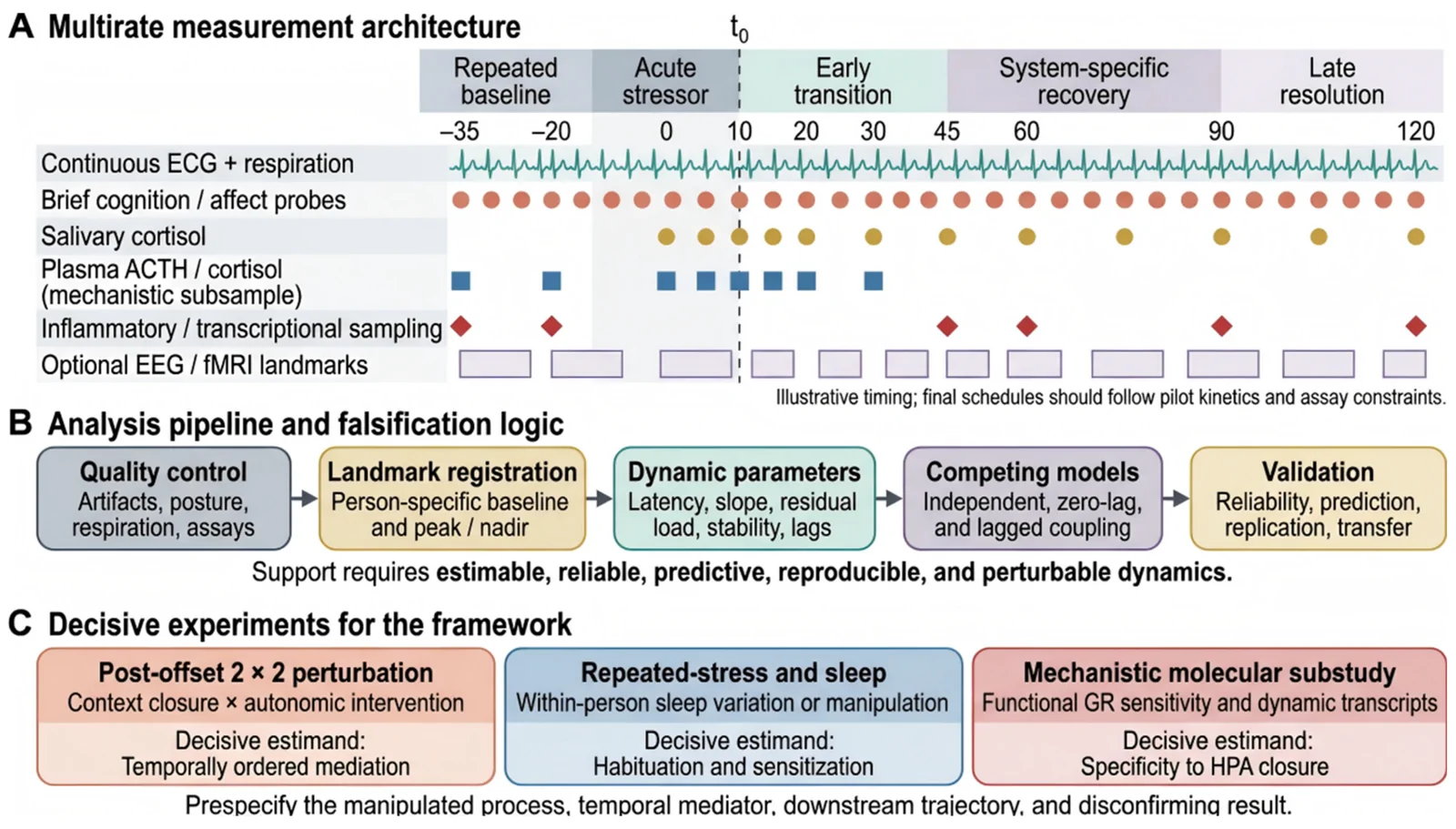
Operationalizing the post-stressor transition. Panel (**A**) distinguishes continuous ECG/respiration, brief cognitive probes, salivary cortisol, venous ACTH/cortisol, and optional immune, transcriptional and neural observations. The timeline is schematic and does not imply that every modality is required or that every indicated time is adequate for every parameter. Table 4 and Section 9 distinguish the planning floor from a validated design. Panel (**B**) contrasts shared-input, independent and coupled explanations after measurement modeling; Panel (**C**) identifies factorial, repeated-stress and molecular tests. No simulation-based reliability or power result is represented. ACTH, adrenocorticotropic hormone; ECG, electrocardiography; EEG, electroencephalography; fMRI, functional magnetic resonance imaging; GR, glucocorticoid receptor; HPA, hypothalamic–pituitary–adrenocortical.

Figure 5 maps multirate measurement to model comparison. Its six measurement rows are alternatives chosen by the estimand, not requirements to collect every modality. Table 4 provides a literature-informed planning floor; it does not guarantee reliable estimation or prescribe one schedule for all participants. Section 9.1, Section 9.2, Section 9.3 and Section 9.4 distinguish observable recovery, model identification, and statistical power.

### 9.1. Sampling Floors and Parameter Identifiability

For a simple salivary-cortisol rise/peak/decline analysis, a practical planning floor is two seated prestressor samples, an offset sample, several samples bracketing the expected peak, and at least three observations after a demonstrable peak. For a 15-min challenge ending at t_0_, an illustrative schedule is −35 and −20 min, t_0_, and +10, +20, +30, +45, +60, +90, and +120 min. Both prestressor samples must precede the actual induction period; we move them earlier for longer paradigms. These are literature-informed proposals, not an empirically established minimum number. Three post-peak points may permit a simple slope but cannot identify a complex individual delay model. Late peaks require extended observation rather than forced placement within a fixed window [7,8,9,11,12].

For cardiac dynamics, continuously record ECG and respiration through baseline, challenge, and recovery. Prespecify approximately stationary five-minute segments for conventional short-term HRV, adding shorter windows only when their bias and reliability have been validated for the chosen metric and protocol [27,59,60]. Overlapping windows do not create independent observations. For subjective state, brief probes at t_0_, +5, +10, +20, +30, +45, +60, and +90 min provide an initial multirate design; eight observations do not by themselves support individual reciprocal-lag estimation. Pooling repeated sessions and hierarchical information, or a denser validated schedule, may be required. Probe reactivity must be evaluated as described in Section 4.3.

The number of participants cannot compensate for inadequate within-person timing when the target is an individual peak or lag. Before confirmatory use, simulate the intended observation times, assay noise, autocorrelation, nonresponse, missingness and plausible kinetic variation. Compare reduced and full schedules for parameter bias, interval coverage, convergence, effect detection, and model-selection error. Section S3 supplies a prospective simulation specification; no simulation-derived minimum sample size or validated power claim is reported here [117].

### 9.2. Nonresponse, Censoring, and Uncertain Extrema

A valid but small cortisol response should ordinarily remain in the primary analysis. Model amplitude and trajectory continuously, with person-level uncertainty, rather than discard participants using an outcome-dependent cut-off. Miller et al. evaluated response-classification rules, including increases of 1.5 nmol/L or 15.5%, in particular panel designs [118]. Such cut-offs can support prespecified sensitivity analyses; they are not universal definitions of stress exposure, resilience, or a distinct transition subtype.

When amplitude is indistinguishable from noise, half-recovery time and percentage recovery are undefined or poorly identified, not zero. Retain those participants for whole-trajectory and other-system analyses, and report recovery-specific estimates only where estimable, with an explicit denominator. A peak at the final sample is right-censored with respect to later recovery. Flat or monotonically declining trajectories may reflect weak induction, anticipatory elevation, timing, or physiological variation. Mixture or hurdle models can be secondary comparisons when well identified, but absence of a response does not establish a discrete biological class. Exclusions should be restricted to prespecified protocol or signal-quality failures and documented separately.

### 9.3. Statistical Comparison of Competing Dynamic Accounts

Specify an observation model before testing Equation (1). M0 contains independent system-specific dynamics with the same measured task input, baseline trends, and assay or sensor filters used in the alternatives. M1 adds a contemporaneous shared input or common factor, allowing different response kinetics without directional links. M2 adds cross-system drift terms; continuous-time coupling can generate lagged association even when explicit delays are zero [119]. M3 adds nonzero delays only if the sampling scheme separates transport, common-input and interaction delays. A delayed association that disappears after sensor kinetics are represented is not evidence for a neural-to-endocrine pathway.

For maximum-likelihood fits to the same observed data and likelihood, report log likelihood, parameter count, AIC and BIC, together with residual dependence and trajectory-level diagnostics. Likelihood-ratio tests apply only to appropriate nested models; boundary or weak-identification cases require calibrated, for example parametric-bootstrap, reference distributions. For Bayesian fits, report prior sensitivity, posterior predictive checks, and held-out log predictive density. Primary comparisons should withhold entire participants and, for transfer claims, sessions or cohorts; tuning remains inside the training partition. Held-out error for a prespecified downstream outcome tests utility beyond fitting the sampled curves. Models with different observations or likelihood scales cannot be compared by raw AIC/BIC values.

Power concerns the chosen estimand, not the number of plotted time points. Design simulations should include the null common-input model as well as meaningful couplings, generating and fitting all candidate models under the actual multirate schedule [117]. Sample size and schedule are accepted only after prespecified precision, coverage, false-positive and power targets are met. An aim of 80–90% power is a design choice, not a guarantee supplied by this review. Report parameter recovery and Monte Carlo uncertainty as well as power. Temporal precedence and improved fit remain associational unless linked to an identified intervention or other strong causal design.

### 9.4. Reporting and Validation

Report all stressor-component times, recovery instructions and probes, posture and breathing conditions, sampling relative to t_0_ and the individual extremum, assay or sensor failures, nonresponse and censoring, and the estimable denominator for each parameter. Report the model and effect bound specified before analysis, all tested moderators, and sensitivity to peak estimation, respiratory adjustment and missing-data assumptions. Return-to-reference should be shown with uncertainty, not inferred from a nonsignificant difference from baseline.

Repeated sessions are required to separate rank-order reliability from mean habituation. Prospective prediction should propagate parameter uncertainty and use a comparator containing baseline, peak response, relevant symptoms and shared inputs. A multimodal claim cannot be supported by selecting one significant endpoint from each domain. A model that fails to generalize across stressors should be described as protocol-specific rather than a general recovery phenotype.

## 10. Phase-Specific Experimental and Intervention Windows

Post-offset manipulation separates the intervention from the external induction period, but not necessarily from continuing biological response generation. Cortisol may still be rising when the manipulation begins. Analyses must therefore test effects on both peak amplitude/timing and subsequent decline rather than automatically labeling every post-offset effect “recovery-specific.” This distinction is particularly important when comparing a context-closure intervention with a slower neuromodulatory or endocrine response.

Figure 4 places candidate interventions on a qualitative timeline. Early context information probes threat updating; respiratory or autonomic manipulations probe cardiovascular dynamics; prefrontal stimulation probes distributed neural control. Later molecular and immune sampling characterizes consequences rather than constituting a treatment. No optimal clinical treatment window has been established by these schematics.

### 10.1. Context-Closure and Cognitive Interventions

A rigorous context-closure intervention should manipulate information rather than reassurance alone. It could explicitly terminate further evaluation, resolve uncertainty about hidden consequences, and separate past performance from current safety. A matched control should receive equal experimenter contact and semantic content without resolving the critical uncertainty. Repeated measures should test whether perceived current threat changes before physiology.

Cognitive strategies may also reduce perseverative processing, but timing and mechanism should be distinguished. Reappraisal, attentional redeployment, memory contextualization, and acceptance can produce similar symptom changes through different processes. A phase-specific trial should prespecify whether the intervention is expected to change threat-state inference, verbal rumination, interoceptive interpretation, or behavioral disengagement. The selected mediator should be measured at a frequency capable of preceding the downstream outcome.

### 10.2. Autonomic Interventions

Slow breathing and HRV biofeedback perturb cardiorespiratory dynamics, whereas taVNS introduces a distinct neuromodulatory input. A taVNS assignment should not be equated with demonstrated cardiac vagal engagement. The primary test is whether the intended intermediate process changes and precedes a prespecified downstream endpoint. Respiratory control and adjustment follow the estimand-specific approach in Section 4.4, not a universal “corrected HRV” rule.

Baseline-dependent benefit is a testable possibility, not a settled dosing principle. A study should predefine whether the expected interaction concerns initial vmHRV, threat appraisal, or spontaneous recovery efficiency, and use continuous rather than data-driven subgroup contrasts. A well-powered equivalent interaction or an adequate state-independent model would weaken the proposed moderation.

### 10.3. Prefrontal Neuromodulation and Combination Designs

Post-stress prefrontal stimulation provides preliminary evidence that cortisol recovery can be altered after the response has been induced [93], but target specificity and mediation remain unresolved. Concurrent EEG or fMRI, ECG, respiration, and cortisol sampling can establish whether neural state change precedes peripheral effects. Trait-dependent theta-burst findings further support prespecified moderator or stratified analyses rather than reliance on one average treatment effect [96].

A 2 × 2 factorial trial crossing context-closure information with an autonomic intervention provides separate estimates of main effects and interaction. Additivity alone does not demonstrate independent biological routes, and an interaction does not identify its mediator. Matched attention and sensory controls, manipulation checks, and temporally ordered outcomes are needed to distinguish architecture from nonspecific intervention effects. The trial directly tests the prespecified paths only when those identification conditions are met.

## 11. A Staged Empirical Program for Testing the PSTM

Table 5 stages the empirical tests from identifiability to prospective transfer. Existing data can establish whether the proposed parameters are observable; they cannot validate a pathway that the original timing did not measure. The prospective platform follows Section 9, with target-specific modalities rather than mandatory maximal measurement.

Each stage has a separate failure interpretation. A nonidentifiable parameter calls for redesign, a precise equivalent effect weakens the specified hypothesis, and failure of external prediction limits generalization. Feasibility should therefore precede mechanistic labeling and clinical use.

Repeated stress and ambulatory follow-up test whether a laboratory parameter predicts later adaptation rather than merely describing the index session. Molecular substudies should prioritize functional sensitivity or prespecified cell-adjusted transcript modules, and clinical studies should establish incremental value beyond baseline symptoms. Replication is required before naming transition subtypes or recommending personalized intervention windows.

## 12. Boundary Conditions and Alternative Explanations

The framework is not a universal classification of successful stress termination. Uncertain or continuing threat, different task meanings, and developmental stage and hormonal state alter the relevant reference and outcome. The decision process in Section 7.3 therefore permits an indeterminate result and does not assign pathology from slow physiology alone.

The largest evidential gap remains direct identification of cross-system pathways in humans. Sensor delays, shared inputs and sparse endocrine sampling can mimic directional timing. Strong intracellular evidence cannot resolve this gap without a measurement and perturbation design linking it to human trajectories.

The adult-centered, structured narrative search is also selective rather than exhaustive. It cannot establish prevalence of transition profiles, synthesize a recovery effect across incompatible measures, or support universal thresholds. The qualitative evidence judgments are transparent but not a formal risk-of-bias assessment. Publication bias and selective reporting in the underlying studies remain possible.

Catecholamines, endocannabinoids, oxytocin and AVP have been incorporated as mechanisms that can cross the organizing functions (Section 5.7). The three-function structure remains revisable: a competing account with fewer or different mechanisms is preferable if it explains the same observations more reliably or predicts independent outcomes better. No model should be preserved by adding unmeasured pathways after every unfavorable result.

## 13. Conclusions

A complete account of acute psychosocial stress must explain both mobilization and termination. Objective stressor offset does not initiate a common recovery phase: autonomic, endocrine, neural, cognitive, immune, and transcriptional processes follow different kinetics and may remain linked through delayed feedback. The PSTM organizes this interval around frontolimbic context updating, vagal re-engagement, glucocorticoid feedback closure, and delayed immune–transcriptional resolution.

The framework’s claims are conditional. Recovery may add useful information beyond reactivity, and coupling may improve an independent model, but both propositions require prespecified contrasts and informative uncertainty. Physiological return and adaptation are separate conclusions; neither simultaneous normalization nor a low response is a universal target. The proposed decision rules and sampling schedules are starting points for validation, not established diagnostic thresholds.

The next advance will come from tests that can fail: measurement-valid trajectories, identified post-offset perturbations, and replication of effects large enough to matter for a stated outcome. Those tests, rather than the breadth of the conceptual diagram, determine whether the PSTM improves explanation of acute psychosocial stress recovery.

## Figures and Tables

**Table 1:** Evidence map for the principal components of the Post-Stressor Transition Model.

Component	Current Evidence Status	What Is Reasonably Supported	Principal Uncertainty	Role in the PSTM
MR/GR and distributed HPA feedback	Strong mechanistic, moderate human trajectory evidence	Corticosteroid receptors and distributed neural feedback regulate HPA activity across time [18,19,20].	Peripheral cortisol cannot isolate receptor-, cell-, or circuit-specific mechanisms.	Molecular basis of glucocorticoid feedback closure.
FKBP4–FKBP5–SKA2 regulation	Strong cellular/animal, limited direct human recovery evidence	Co-chaperones and SKA2 alter GR sensitivity, trafficking and signaling; direct human recovery evidence remains limited [21,22,23,24].	Tissue specificity, developmental context, and modest human genetic effects.	Candidate regulator of feedback gain, not a master switch.
Dynamic GR-responsive transcription	Strong molecular plausibility, emerging time-resolved human evidence	GR activation and psychosocial stress can alter expression programs, but pharmacological and natural-stress evidence are distinct [25,26].	Cell composition, tissue mismatch, timing, and unclear causal relation to cortisol decline.	Candidate functional bridge from receptor signaling to measurable closure.
Cardiac vagal re-engagement	Moderate but methodologically heterogeneous	Post-stressor vmHRV varies across protocols and may add information to heart-rate measures [27,28,29].	Respiration, posture, mean heart rate, and window selection.	Early peripheral transition and possible feedback channel.
Frontolimbic context updating	Moderate indirect, emerging phase-specific recovery evidence	Stress changes distributed neural states; event-closure directionality remains a candidate interpretation [30,31,32,33,34].	Directionality between neural updating, cognition, and physiology.	Candidate mechanism translating event completion into state change.
Perseverative cognition	Moderate; effects generally small to modest	Worry and rumination are associated with physiological activation; effects and measurement definitions vary [35,36,37].	Between-person confounding, construct overlap, and inconsistent recovery measurement.	Potential internal stressor that delays context updating.
Delayed immune and transcriptional resolution	Strong evidence for delayed circulating inflammatory change; emerging evidence for transition-linked transcriptional resolution.	Repeated blood sampling documents delayed IL-6 and nonidentical endocrine/immune habituation [26,38,39,40].	Cell composition, assay timing, tissue specificity, and adaptive versus harmful meaning.	Delayed downstream and reciprocal layer.
Cross-system lagged coordination	Emerging	Systems have different kinetics; lag alone does not distinguish coupling from shared input or measurement filters.	Reliability, generalizability, and superiority over independent models.	Central falsifiable proposition of the model.
Prospective clinical validity	Insufficient for clinical classification	Selected observational associations motivate prospective testing, not outcome thresholds.	Replication, task generalization, incremental prediction, and clinical utility.	Required before phenotyping or personalization.

Note: Evidence labels are qualitative, design-informed narrative judgments; Section 2 defines their criteria. A strong cellular mechanism does not imply strong evidence for an entire human transition pathway. These are not formal GRADE ratings. GR, glucocorticoid receptor; HPA, hypothalamic–pituitary–adrenocortical; MR, mineralocorticoid receptor; PSTM, Post-Stressor Transition Model; SKA2, spindle and kinetochore-associated protein 2; vmHRV, vagally mediated heart rate variability.

**Table 2:** Core concepts and dynamic measures for post-stressor transition research.

Construct/Metric	Operational Definition	Recommended Estimation	Primary Interpretive Risk
Objective stressor offset (t_0_)	Timed end of the external challenge; any continuing evaluation or uncertainty is recorded separately.	Record each component separately; state whether uncertainty or observation continued.	Nominal task end may not equal social-evaluative end.
System-specific peak/nadir	Peak or nadir within a prespecified episode window, estimated with uncertainty.	Model-implied and observed peaks with sensitivity analyses.	Sparse sampling can place the apparent peak at the final observation.
Offset-relative extremum timing	Signed interval t_extremum − t_0_; it can be negative.	Retain offset and extremum anchors; flag boundary estimates and unobserved later recovery.	Not a direct measure of neural or endocrine termination; “termination latency” can overstate mechanism.
Recovery rate	Post-peak or post-nadir movement toward a relevant reference state.	Piecewise slope, nonlinear decay constant, or continuous-time return parameter.	Depends on peak definition and assumed functional form.
Half-recovery time	Time required to resolve 50% of the peak deviation.	Derived from a nonlinear or person-specific trajectory.	Undefined or unstable when measurable response amplitude is absent or close to zero.
Residual post-stressor load	Exposure remaining after the peak over a prespecified interval.	Peak-adjusted residual AUC or integrated deviation from reference.	Confounds recovery with observation-window length if not standardized.
Time to stability	Time at which the process enters and remains within an individual reference band.	Change-point or state-space estimate with a minimum dwell criterion.	Apparent return can be followed by rebound or overshoot.
Overshoot/rebound	Movement beyond the reference level after initial return.	Signed maximum deviation after first return and subsequent stabilization.	Can be adaptive regulation or measurement artifact.
Cross-system lag	Delay at which change in one system best predicts another.	Continuous-time models, dynamic structural equations, or preregistered lag models.	Shared trends and different sampling rates can create spurious directionality.
Directional coupling	Estimated influence of one evolving process on another.	State-space or coupled differential-equation model compared with independent alternatives.	Association is not causal without perturbation or stronger identification.

Note: AUC, area under the curve; t_0_, objective stressor offset.

**Table 3:** Falsifiable propositions, disconfirming observations, and priority tests.

Confirmatory Claim	Contrast, Direction and Window	Primary Estimand/Comparator	Result That Weakens the Specified Claim
1. Recovery adds predictive information.	Predict a prespecified second-session or prospective outcome from peak-aligned cortisol decline (the individual peak to 60 min after that peak; extend observation if needed).	Held-out ΔR^2^ above baseline, peak, timing and covariates; require gain ≥ δ_pred.	Upper confidence bound below δ_pred in an adequately sampled independent test; an unreliable estimate is inconclusive.
2. Context updating affects later physiology.	Randomize closure information at t_0_; current-threat change at 0–5 min should precede higher log-RMSSD at 5–20 min and/or faster later cortisol decline.	Preselect one downstream contrast of magnitude ≥ δ; model peak effects separately.	Valid manipulation but equivalent downstream contrast within ±δ, or reliable reversed temporal order.
3. Coupling adds beyond shared kinetics.	Prespecify cognitive-to-cardiac lags at 5–10 min unless validated short-window data permit finer timing; endocrine follow-up at 10–60 min; immune outcomes at 60–120 min.	Held-out likelihood/outcome error versus identical observation models with independent or shared inputs.	Coupled model does not improve independent prediction by prespecified δ_pred; common-input/filter model explains the delay.
4. Functional GR indices relate to HPA decline.	Prespecified ex vivo GR sensitivity or transcript module predicts steeper peak-aligned cortisol decline, controlling amplitude; compare cardiac mobilization.	Standardized HPA association ≥ δ and larger than the prespecified cardiac association; assay direction defined in advance.	Equivalent HPA association, opposite direction, or no HPA specificity in an independent sample.
5. Post-event rumination prolongs activation.	Manipulate rumination only after t_0_; higher threat at 0–10 min and larger cortisol residual load over 20–90 min are predicted.	Prespecified downstream contrast ≥ δ, with baseline/peak modeled; probe reactivity controlled.	Manipulation changes cognition but downstream contrast is equivalent within ±δ; a wide interval is inconclusive.
6. Autonomic intervention effects depend on state.	In a dose/state test, lower baseline log-RMSSD or poorer spontaneous stabilization predicts greater 5–30 min change, assessed again off pacing.	Prespecified interaction or nonlinear term; compare linear state-independent alternative.	Adequate state-independent model and interaction equivalent within prespecified bounds across replicated states/doses.
7. Sleep contributes beyond response magnitude.	Within-person sleep efficiency predicts faster peak-aligned cortisol decline over 20–90 min or another single preregistered recovery outcome.	Within-person slope ≥ δ after baseline, amplitude and timing; separate stable average sleep.	Recovery slope equivalent within ±δ, with effect limited to reactivity or not reproducible.
8. Transition predicts later adaptation.	Index-session transition estimates predict a second session and/or event-triggered daily-life recovery at a prespecified follow-up.	Held-out improvement ≥ δ_pred over reactivity and symptoms; one primary adaptation endpoint.	Precise improvement below δ_pred or failure in an independent cohort; descriptive fit is not clinical validity.

Note: Times are minutes relative to objective stressor offset unless indexed to the individual peak. All contrasts, windows, effect bounds, and multiplicity rules must be prespecified. δ denotes a study-specific SESOI; |d| = 0.20 or ΔR^2^ = 0.02 are illustrative planning conventions only, not empirical thresholds. An imprecise null is inconclusive, not disconfirmation. GR, glucocorticoid receptor; HPA, hypothalamic–pituitary–adrenocortical-.

**Table 4:** Literature-informed measurement proposals and identification safeguards for adult post-stressor studies.

Domain	Planning Floor/Candidate Measurements	Illustrative Schedule Relative to t_0_	Identification and Interpretive Safeguard
Context/cognition	Brief current-threat and event-completion probes; distinguish rumination and affect.	t_0_; +5, +10, +20, +30, +45, +60, +90 min.	Eight probes are not a guarantee for person-specific lags; log probe inputs and test sparse/no-probe controls.
ECG/respiration	Continuous ECG, respiratory frequency and relative amplitude; heart rate and log-RMSSD.	Repeated seated baseline, challenge, and ≥60–90 min recovery.	Approximately stationary 5-min HRV windows; shorter windows require protocol-specific validation. Report overlap, artifacts and respiratory sensitivity.
Sympathetic/vascular	Blood pressure, electrodermal activity and pre-ejection period when feasible.	Continuous or frequent early observations plus later matched rest windows.	A heart-rate decline alone does not distinguish sympathetic withdrawal from vagal return.
Salivary cortisol	Two true prestressor samples; offset; peak-bracketing samples; ≥3 observed post-peak points for a simple slope.	Example for a 15-min challenge: −35, −20, 0, +10, +20, +30, +45, +60, +90, +120 min; adapt to actual onset.	Literature-informed planning floor, not a validated minimum. Extend for late peaks; no-response T50 is undefined.
Plasma ACTH/cortisol	Venous sampling in a mechanistic subsample, with standardized collection/processing.	True baseline; late challenge; t_0_; approximately +5, +10, +20, +30 min, then extend as required.	ACTH kinetics and sample handling differ from salivary cortisol; venipuncture/cannulation may alter stress.
Inflammation/transcription	Selected cytokines; leukocyte composition; prespecified transcript module or GR sensitivity.	True baseline; illustrative +30/+45, +90, +120 min; later sampling if decline is the target.	Cytokine rise is not resolution. Transcription and cytokine schedules need separate pilot kinetics.
EEG/fMRI	Matched rest or brief probes; prespecified spectral, state or connectivity outcomes.	Prestressor, immediate post-offset, and selected later landmarks.	Motion/eye-state control; scalp connectivity does not identify deep limbic causality.
Development/hormonal state	Age; pubertal stage when relevant; menstrual phase method, contraception, menopause and hormonal therapy.	Baseline metadata; state verified for each repeated session where feasible.	Report even without interaction power. Test measurement/gain differences before recovery-specific effects.
Plasma ACTH/cortisol	Venous sampling in a mechanistic subsample, with standardized collection/processing.	True baseline; late challenge; t_0_; approximately +5, +10, +20, +30 min, then extend as required.	ACTH kinetics and sample handling differ from salivary cortisol; venipuncture/cannulation may alter stress.
Sleep/validation	Several nights of actigraphy/diary; repeated stress; ambulatory ECG/EMA and prospective outcomes.	Before index session, repeat session, then prespecified follow-up.	Model within-person sleep deviations separately; hold out participants/sessions; validate independent functional outcomes.

Note: These schedules are planning proposals informed by the cited measurement and stress literature, not simulation-validated minima or a mandatory multimodal protocol. Baseline samples must precede the actual stressor. Reliable estimation depends on signal quality, within-person timing, sample size and the target model. ACTH, adrenocorticotropic hormone; ECG, electrocardiography; EEG, electroencephalography; EMA, ecological momentary assessment; fMRI, functional magnetic resonance imaging; GR, glucocorticoid receptor; HRV, heart rate variability; RMSSD, root mean square of successive differences; T50, half-recovery time; t_0_, objective stressor offset.

**Table 5:** Staged empirical program for testing the Post-Stressor Transition Model.

Stage	Core Study	Decisive Estimand	Predefined Success Criterion	Main Failure Mode and Interpretation
1. Feasibility	Reanalysis of existing stress datasets with individual peak registration.	Identifiability of extrema and post-extremum parameters; reliability only when repeated data exist.	Parameters are estimable with uncertainty and are not forced to observation boundaries.	Sparse sampling prevents identification; informs redesign rather than supporting a mechanism.
2. Platform	Prospective 90–120 min multimodal post-stressor protocol.	Session reliability and incremental information beyond baseline and peak.	Key parameters show usable uncertainty and repeatability across sessions.	Parameters are task- or day-specific; narrow the target or increase measurement precision.
3. Perturbation	Post-offset 2 × 2 factorial design: context closure × autonomic intervention.	Temporally ordered mediation and change in coupling.	The targeted intermediate changes before a prespecified downstream outcome under an identified contrast.	Imprecise null: inconclusive; precise equivalent or contradictory path: specified mechanism weakened.
4. Adaptation	Repeated-stress and sleep design.	Prediction of habituation, sensitization, and day-to-day stability.	Initial transition parameters predict later adaptation beyond reactivity.	No prospective signal; parameters may be descriptive rather than adaptive traits.
5. Transfer	Ambulatory, cross-diagnostic, and mechanistic molecular validation.	Ecological prediction, replication, and specificity to HPA closure.	Held-out prediction and mechanistic selectivity replicate in an independent sample.	No transfer or specificity; avoid clinical labels and retain protocol-specific interpretation.

Note: HPA, hypothalamic–pituitary–adrenocortical.

## Data Availability

No new data were created or analyzed in this study. Data sharing is not applicable to this article.

## Supplementary Materials

The following supporting information can be downloaded at: https://www.mdpi.com/article/10.3390/ijms27209047/s1.

## Abbreviations

ACTH	adrenocorticotropic hormone
AIC	Akaike information criterion
AP-1	activator protein 1
AUC	area under the curve
AVP	arginine vasopressin
BIC	Bayesian information criterion
CI	confidence interval
DLPFC	dorsolateral prefrontal cortex
ECG	electrocardiography
EMA	ecological momentary assessment
FKBP	FK506-binding protein
GR	glucocorticoid receptor
GRE	glucocorticoid response element
HPA	hypothalamic–pituitary–adrenocortical
HRV	heart rate variability
IL-6	interleukin-6
MR	mineralocorticoid receptor
NF-κB	nuclear factor kappa B
PSTM	Post-Stressor Transition Model
RMSSD	root mean square of successive differences
SESOI	smallest effect size of interest
SKA2	spindle and kinetochore-associated protein 2
t_0_	objective stressor offset
taVNS	transcutaneous auricular vagus nerve stimulation
TSST	Trier Social Stress Test
vmHRV	vagally mediated heart rate variability
