# Supplementary material for: How the Acute Psychosocial Stress Response Ends: A Post-Stressor Transition Model of Glucocorticoid Feedback, Vagal Re-Engagement, and Frontolimbic Context Updating
Source: Int J Mol Sci. 2026 Oct 12;27(20):9047. doi: 10.3390/ijms27209047 (PMC13704977; doi:10.3390/ijms27209047)
Supplement: Supplementary file 1 [file ijms-27-09047-s001.zip › ijms-4584614-supplementary.pdf]

Supplementary Methods

S1. Evidence identification and scope

PubMed/MEDLINE was the only bibliographic database directly consulted. The evidence base reported in the submitted manuscript extended through 10 August 2026. Targeted revision-stage retrieval in September 2026 addressed the specific topics below, using PubMed records and linked accessible full texts. Backward and forward citation tracking supplemented record retrieval.

The review is structured and narrative: its purpose is to compare mechanisms and testable interpretations, not estimate a pooled recovery effect or enumerate every eligible study. There was no preregistered systematic-review protocol, formal duplicate screening, or formal GRADE assessment. An exact historical search-yield ledger was not available in the supplied materials; no retrospective record counts, PRISMA flow diagram, or reconstructed screening totals are reported.

S1.1. Targeted revision retrieval

The revision added records on: earlier conceptual accounts of physiological recovery; updated HRV measurement recommendations; cortisol responder classification; repeated inflammatory and transcriptional measurements; developmental and reproductive-hormonal variation; endocannabinoid, oxytocin and vasopressin/copeptin findings; continuous-time models; simulation-study design; and equivalence testing. Exact-title retrieval and DOI/PMID checks were used to verify the added bibliography. Only published journal articles were retained; preprints and database-only title matches without confirmable publication details were not added.

Representative verified anchor records include Linden et al. (1997; PMID 9076640), Quigley et al. (2024; PMID 38873876), Miller et al. (2013; PMID 24184845), von Känel et al. (2006; PMID 15890495), McInnis et al. (2014; PMID 25107874), McInnis et al. (2015; PMID 25683696), Hill et al. (2011; PMID 21775596), and Lakens (2017; PMID 28736600). The added-reference audit provides the complete set of identifiers.

S1.2. Reproducible topic blocks for future updates

The independent blocks below operationalize the narrative scope for future PubMed updates.

("Stress, Psychological"[Mesh] OR "psychosocial stress"[tiab] OR "psychological stress"[tiab] OR "Trier Social Stress Test"[tiab]) AND (recover*[tiab] OR "post-stress"[tiab] OR "post-stressor"[tiab] OR habituat*[tiab])

("psychosocial stress"[tiab] OR "psychological stress"[tiab] OR "Trier Social Stress Test"[tiab]) AND (cortisol[tiab] OR glucocorticoid*[tiab] OR FKBP5[tiab]) AND (sampling[tiab] OR peak[tiab] OR nonrespond*[tiab] OR recover*[tiab])

("psychological stress"[tiab] OR "psychosocial stress"[tiab]) AND ("heart rate variability"[tiab] OR vagal[tiab] OR autonomic[tiab]) AND (respirat*[tiab] OR recover*[tiab] OR "measurement guidelines"[tiab])

("psychological stress"[tiab] OR "psychosocial stress"[tiab]) AND ("interleukin-6"[tiab] OR cytokine*[tiab] OR transcript*[tiab] OR "gene expression"[tiab]) AND (repeated[tiab] OR habituat*[tiab] OR sampling[tiab] OR recover*[tiab])

(stress[tiab]) AND (endocannabinoid*[tiab] OR anandamide[tiab] OR oxytocin[tiab] OR vasopressin[tiab] OR copeptin[tiab]) AND (recover*[tiab] OR termination[tiab] OR "Trier Social Stress Test"[tiab])

("psychosocial stress"[tiab] OR "psychological stress"[tiab]) AND (puberty[tiab] OR adolescen*[tiab] OR aging[tiab] OR elderly[tiab] OR "menstrual cycle"[tiab] OR contraceptive*[tiab]) AND (cortisol[tiab] OR autonomic[tiab] OR recover*[tiab])

S1.3. Evidence interpretation

Claims were separated by species, intervention timing, measurement compartment and design. Pharmacological GR-response studies were not treated as direct psychosocial-recovery studies; peripheral biomarkers were not treated as central measures; pre-stressor interventions were not treated as isolated recovery manipulations. Table 2 uses five narrative criteria—design, replication, temporal resolution, measurement specificity and direct relevance—without converting these criteria into a numerical score.

S2. Operational decision rules and confirmatory tests

S2.1. What “adaptive” can and cannot mean

First determine whether the episode and data are interpretable: external conditions, perceived continuing evaluation, prestressor state, sensor quality, sampling coverage and the extremum must be documented. If the peak or descending limb was not observed, report this

directly rather than infer recovery from the last sample. Next describe each system continuously and classify only within the observation window: resolved, unresolved, or indeterminate. Any reference band and dwell criterion must be defined independently of the outcome data and adjusted to the protocol and assay.

A 95% prediction band estimated from matched no-stress observations and a requirement for two consecutive observations within it are examples of operational conventions, not normative cut-offs. A band based on only two baseline samples is generally not sufficient to estimate an individual time-varying reference distribution. Closely overlapping HRV windows must not be counted as independent dwell observations. Return to a band is descriptive; an adaptive interpretation additionally requires a prespecified, independent favorable outcome without a relevant trade-off.

S2.2. Prespecifying a decisive effect

For each proposition in Table 3, prespecify the exposure or intervention, primary endpoint, baseline and peak adjustments, observation window, direction, scale, SESOI, missing-data assumptions and multiplicity procedure. The example standardized contrast $|d| = 0.20$ and held-out $\Delta R^2 = 0.02$ illustrate an auditable design decision only. Their use must be justified against measurement reliability, the cost or benefit of the outcome, prior credible evidence and feasible precision. They are not model-estimated effects or universal clinical thresholds [111].

For a directional benefit, define its sign before analysis and assess whether a confidence bound excludes the minimum meaningful benefit. For equivalence, use prespecified lower and upper bounds; two one-sided tests at $\alpha = 0.05$ correspond to a 90% confidence interval entirely inside those bounds. A nonsignificant conventional test with a wide interval is not evidence of equivalence. Report effect size and uncertainty even when the conclusion is inconclusive [111].

For prediction, define the comparator and target outcome in advance. A change in held-out R^2 should be calculated on the same outcome and test partition, with its uncertainty propagated over participants; it is not an in-sample increase obtained by adding parameters. For likelihood-based comparison, use the same observations and observation model. Do not switch between cortisol, HRV and affect after inspecting significance.

S2.3. A worked design specification—not an executed study

One test of Proposition 2 would randomize event-closure information immediately after

the externally timed task end. The primary manipulation check would be current-threat appraisal during 0–5 minutes; the primary downstream contrast could be respiration-aware log-RMSSD during a prespecified 5–20-minute interval. Cortisol peak amplitude/timing and post-peak decline would be distinct secondary outcomes. Matched experimenter contact, no-speech measurement windows and a sparse-probe control would help separate semantic information, breathing and assessment effects. A valid manipulation combined with an equivalent downstream effect within justified bounds would weaken this specified pathway; poor manipulation or insufficient precision would remain inconclusive.

S3. Sampling, competing models and prospective simulation specification

S3.1. Literature-informed sampling versus validated minimum

The schedule in Table 4 draws on laboratory stress and cortisol-modeling work, conventional short-term HRV recommendations and repeated inflammatory sampling [7–9,11,12,45–47,104–106]. It is a planning floor, not proof that a given number of samples is sufficient. A simple post-peak slope and an individual delayed-coupling model require different information. Valid nonresponders remain in the primary whole-trajectory analysis; percentage recovery and half-recovery time are not assigned zero when amplitude is absent [118].

A common offset timeline must be retained when individually registering peaks: registration must not erase the delay that the coupling model is intended to estimate. For comparisons across modalities, make their different observation filters explicit, including HRV window averaging and saliva transport. Do not align each series independently and then interpret the resulting artificial synchrony as biological coordination.

S3.2. Candidate models

M0: independent latent system dynamics conditional on the same observed task inputs, baseline trends and measurement filters. M1: add a shared dynamic input or factor, with separate system response kinetics but no direct cross-system effects. M2: add cross-system drift terms; the continuous-time system can yield lagged association without an explicit delay. M3: add a small, prespecified set of explicit delays only after demonstrating that the actual design identifies them separately from shared inputs and sensor filters [119].

Use the same outcome samples and compatible likelihoods across candidates. Report

likelihood and parameter count, AIC/BIC for appropriate maximum-likelihood fits, residual autocorrelation and posterior/parametric predictive checks. Nested likelihood tests require valid reference distributions; use calibration when delays are on boundaries or poorly identified. Primary utility should be tested by withholding participants and, for generalization, sessions or cohorts. AIC or BIC superiority alone does not show causality or practical value.

S3.3. Prospective ADEMP-style simulation plan

No simulation has been executed for this review. The specification below is supplied to guide study-specific design validation, following the principles of explicit aims, data-generating mechanisms, estimands, methods and performance measures [117]. It must not be cited as evidence that a particular sample size has established power.

Aim: quantify whether the planned schedule can distinguish independent, common-input and coupled dynamics, and estimate the primary intervention or prediction contrast. Data generation: vary plausible baseline/gain distributions, early and late peaks, state return rates, zero and nonzero coupling, observation delays, autocorrelation, assay noise, weak responses and missingness. Use pilot or external estimates where available; distinguish those from analyst-chosen stress-test values.

An illustrative design grid may consider N = 100, 200, 400 and 800; one versus repeated sessions; the complete schedule versus reduced peak-bracketing or late sampling; and 0%, 10% and 20% missing observations. These numbers are hypothetical grid points, not recommendations or results. Include both missing-at-random and plausible informative missingness scenarios, and evaluate separate loss of an entire participant versus isolated samples.

Fit the planned models under the same preprocessing and participant-level validation pipeline. Record convergence and boundary solutions, bias and root mean squared error of each target parameter, interval coverage, Type I error under the common-input null, power for the prespecified SESOI, and false selection of a coupled model. An 80–90% power target can be chosen prospectively, but parameter coverage and false positives must also be acceptable. Increase replicates until Monte Carlo uncertainty for the primary performance measure meets a declared tolerance [117].

Report all failed fits and nonidentifiable parameters rather than calculating power only

among successful fits. If M3 delays remain weakly identified, retain a simpler M1 or M2 as the confirmatory model instead of increasing complexity. A larger between-person sample does not reconstruct an unobserved within-person peak.

Table S1. Relation of the PSTM to preceding accounts

Prior account	Existing contribution credited	Operational addition proposed here	Test required before claiming added value
Recovery / peak-adjusted analysis [10,12,13]	Separates reactivity and recovery; estimates nonredundant endocrine parameters.	Retain both objective offset and individual extrema, linked to phase-specific mechanisms.	Added prediction or identified perturbation beyond the same baseline/peak model.
Multi-trajectory analysis [14–17]	Describes heterogeneity and cross-system covariation.	Compare common-input and directional models rather than assign mechanism to a class.	Independent validation and discrimination between shared kinetics and coupling.
Allostasis [1,2]	Explains context-dependent regulation and cumulative biological cost.	Specifies a minutes-to-hours observation window and outcome-based interpretation.	Prospective effects beyond index-session physiology; no claim to replace allostasis.
Neurovisceral integration [48,49]	Links central regulation to autonomic flexibility.	Adds endocrine timing, measurement filters and post-offset perturbation contrasts.	Respiration-aware intermediate change preceding a specified downstream effect.
Perseverative cognition [56,57]	Explains activation beyond direct external exposure.	Separates continuing threat, event completion and repetitive thought; tests probe reactivity.	Information/rumination intervention with temporally ordered, independently selected outcomes.

References in this supplement follow the revised main manuscript. The table compares theoretical scope, not empirical superiority.
